# Mapping the stability of febrile illness hotspots in Punjab from 2012 to 2019- a spatial clustering and regression analysis

**DOI:** 10.1186/s12889-023-16930-y

**Published:** 2023-10-16

**Authors:** Madhur Verma, Shweta Panwar, Soumya Swaroop Sahoo, Gagandeep Singh Grover, Seema Aggarwal, Jaya Prasad Tripathy, Jitendra Shah, Rakesh Kakkar

**Affiliations:** 1 Department of Community and Family Medicine, All India Institute of Medical Sciences, Bathinda, Punjab India; 2 Centre for Technology Alternatives for Rural Areas (CTARA), Indian Institute of Technology Bombay, Mumbai, Maharashtra India; 3 Directorate of Health and Family Welfare Punjab and State Programme Officer, Integrated Disease Surveillance Program Punjab, Chandigarh, India; 4 Department of Community and Family Medicine, All India Institute of Medical Sciences, Nagpur, Maharashtra India

**Keywords:** Vector-borne disease, Infectious disease epidemiology, Geospatial analysis

## Abstract

**Introduction:**

Febrile illnesses (FI) represent a typical spectrum of diseases in low-resource settings, either in isolation or with other common symptoms. They contribute substantially to morbidity and mortality in India. The primary objective was to study the burden of FI based on Integrated Disease Surveillance Programme (IDSP) data in Punjab, analyze geospatial and temporal trends and patterns, and identify the potential hotspots for effective intervention.

**Methods:**

A retrospective ecological study used the district-level IDSP reports between 2012 and 2019. Diseases responsible for FI on a large scale, like Dengue, Chikungunya, Malaria (Plasmodium *Falciparum*, P. *Vivax*), Enteric fever, and Pyrexia of Unknown Origin (PUO), were included in the analysis. The digital map of Punjab was obtained from GitHub. Spatial autocorrelation and cluster analysis were done using Moran’s I and Getis-Ord G* to determine hotspots of FI using the incidence and crude disease numbers reported under IDSP. Further, negative binomial regression was used to determine the association between Spatio-temporal and population variables per the census 2011. Stable hotspots were depicted using heat maps generated from district-wise yearly data.

**Results:**

PUO was the highest reported FI. We observed a rising trend in the incidence of Dengue, Chikungunya, and Enteric fever, which depicted occasional spikes during the study period. FI expressed significant inter-district variations and clustering during the start of the study period, with more dispersion in the latter part of the study period. P.*Vivax* malaria depicted stable hotspots in southern districts of Punjab. In contrast, P. *Falciparum* malaria, Chikungunya, and PUO expressed no spatial patterns. Enteric Fever incidence was high in central and northeastern districts but depicted no stable spatial patterns. Certain districts were common incidence hotspots for multiple diseases. The number of cases in each district has shown over-dispersion for each disease and has little dependence on population, gender, or residence as per regression analysis.

**Conclusions:**

The study demonstrates that information obtained through IDSP can describe the spatial epidemiology of FI at crude spatial scales and drive concerted efforts against FI by identifying actionable points.

**Supplementary Information:**

The online version contains supplementary material available at 10.1186/s12889-023-16930-y.

## Introduction

Febrile illness (FI) is a nonspecific manifestation of infectious diseases characterized by malaise, myalgia, and a raised temperature [1]. They are a common cause of outpatient visits and hospital admissions, contributing considerably to morbidity and mortality in Low- and Middle-Income countries, including India, where the burden of infectious diseases is concerning [2–4]. India has been home to episodic outbreaks of FI in recent decades; most of them were attributed to emerging and re-emerging Vector-borne diseases (VBD) [5]. Many preventable deaths occur due to these FI because of their varied presentation, leading to delayed diagnosis and untimely access to adequate health care and laboratory facilities, particularly in low-resource settings [6]. FI's diagnosis and clinical decision-making can be challenging without evidence-based epidemiological data in the Indian subcontinent [7].

The global capacity to respond promptly to potentially epidemic-prone FIs depends greatly on increased readiness, efficient surveillance, and monitoring systems [8]. However, the lack of research in areas of infectious disease epidemiology has restrained the development of such systems, especially in resource-constrained settings from where most of the FI outbreaks and epidemics are reported [9]. This has been exemplified by the enormous difficulties faced while managing the COVID-19 pandemic, further stimulating us to bolster our surveillance systems [10]. Since the deadly disease outbreaks in the early 2000s, the Government of India stringently augmented its efforts to substantiate its epidemic-prone infectious disease surveillance and response system [11]. One of the significant interventions was implementing an integrated disease surveillance program (IDSP) under the National Centre for Disease Control in 2004. This program aims to monitor infectious diseases, including FIs, and respond to them with minimum reaction time [12]. Still, IDSP has challenges, like a significant lag time and incomplete or inappropriate reporting by peripheral health workers [13]. Further, the use of this evidence-based epidemiological surveillance data generated by IDSP for a systematic approach to the cause of FI and appropriate management in Indian states is limited primarily to descriptive epidemiology.

Like other states of India, FIs pose a significant public health problem in Punjab, a state of India, and frequently experiences spikes in FI. Such spikes can be broadly attributable to factors like an agrarian economy, sizeable livestock population, dense housing patterns, inward migration, rapid urbanization, and the growth of slums [14, 15]. Previous analyses from Punjab have depicted Malaria (Plasmodium *Vivax* and Plasmodium *Falciparum*), Dengue, Chikungunya, Enteric fever, and some diseases conditions classified as Pyrexia of Unknown Origin (PUO) are the most common causes of FI in Punjab [16]. Malaria cases have the highest prevalence in the districts across the western border, while Dengue and Chikungunya were prominent towards the southeastern borders [16]. Malaria and Chikungunya depicted significant urban–rural and gender disparities, with striking temporal trends [16]. A hospital-based study from Chandigarh, the capital of Punjab, depicted the incidence of Enteric fever to be 1622 cases per 100,000 child-years among children between the ages of 6 months and 14 years and 970 cases per 100,000 person-years among those who were 15 years of age or older [17]. The cohort component of the same study depicted the national incidence rates of Enteric fever to be around 1.73 (1.72–1.74) per child per year of observation [17]. Another Modelling study predicted the incidence rates to be about 427 (353–580) cases per 100,000 person-years [18]. The PUO was first defined in 1961 but remains a clinical challenge for many physicians, and hence, the epidemiological burden in Punjab is still hard to estimate; most of the literature comes from hospital-based studies and mostly targets etiology [19]. However, in a systematic review of literature mainly examining retrospective trial data, Fusco et al. reported that the overall incidence of PUO ranged from 8.5% to 51.0% [20].

With such a burden, it would be essential to know from where to start and which districts maximally contribute to the FI disease burden of the state so that targeted interventions can be implemented to contain the disease spread. It has been observed that FI outbreaks show clustering of cases concerning area and populations, which mandates a better understanding of the spatial epidemiology of FI [21, 22]. The transmission of FI often shows substantial spatial heterogeneity that challenges disease containment [23]. Within this context, FI hotspots are defined as areas of persistently elevated disease burden or where the transmission intensity exceeds average levels [24]. Hotspots have been proposed as reservoirs of residual transmission, which may perpetuate the spread to larger areas with time, and mapping hotspots can help us localize foci of transmission and possibly the socio-behavioral and environmental factors determining disease spread [25]. Therefore, identifying transmission clusters or hotspots can provide an opportunity to engage in targeted infectious disease control. However, identifying hotspots remains challenging because of the interplay between ecological conditions and vector factors [23]. Hotspot analysis has the necessary potential, as per experts worldwide [9, 18, 22, 25–28]. Therefore, the present study aims to identify stable hotspots across different districts of Punjab state. The primary objective is to analyze temporal trends and geospatial patterns based on the IDSP data of Punjab from 2012–19. Results from such a study may augment surveillance and targeted interventions of these potential hotspots, providing an opportunity for cost-effective and judicious utilization of limited resources.

## Methods

### Study design and setting

**Fig. 1:**
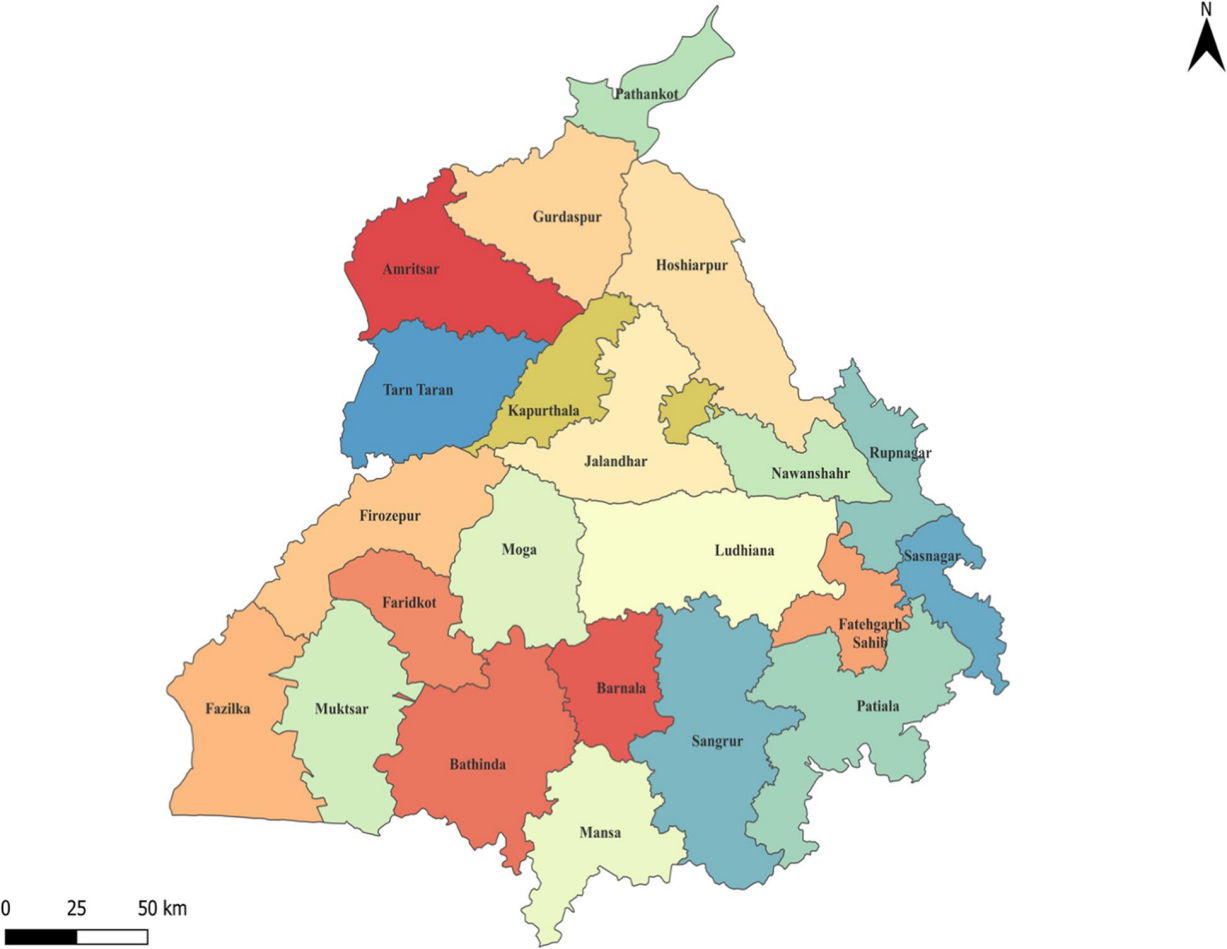
Map of Punjab (India) depicting the geographical boundaries of different districts from where the IDSP was reported during the study period

This study employs a retrospective ecological design to analyze secondary data from the Integrated Disease Surveillance Program (IDSP) in Punjab, India, a northern mid-sized state that shares boundaries with Pakistan. Punjab, the 16th largest state by population, encompasses around 50,000 square kilometers and is divided into 22 districts (Fig. 1). These districts feature diverse terrains, rich irrigated lands, and varied geography, including semi-urban and urban areas, making the region conducive to Vector-borne diseases (VBD) and FI [29]. The state is actively working towards eliminating VBD through the National Vector Born Disease Control Program (NVBDCP), which addresses six major vector-borne diseases i.e., Malaria, Dengue, Chikungunya, Japanese Encephalitis, Lymphatic Filariasis, and Kala Azar, with the support of public health systems and private health facilities [30].

### Data source

Data for this study was sourced from the IDSP portal of Punjab [12]. Twenty-two diseases, any other State-Specific Diseases, and any unusual Syndromes not captured above are under usual surveillance. Of these, 12 are reported through laboratory confirmation*.* The state surveillance unit under IDSP receives weekly updates about these diseases via the district surveillance units through ‘S’ (suspected cases), ‘P’ (presumptive cases), and ‘L’ (laboratory-confirmed cases). The health workers fill out the ‘S’ form to report data on suspected cases/syndromes. Medical Officers fill up the ‘P’ form to report data on probable/clinically suspected cases and the ‘L’ form is designed to collect data on lab-confirmed cases. This weekly data provides critical information on disease trends and seasonality patterns. Respective State/District Surveillance Units are undertaking data analysis and actions, later compiled at the central level. Any surge in illness trends prompts investigations by Rapid Response Teams (RRT) for outbreak diagnosis and control [12].

Data types:

- 1. Febrile Illness Data: District-level IDSP reports from 2012 to 2019 were meticulously analyzed to include major FI prioritized by NVBDCP (Dengue, Chikungunya, Malaria (P. *Vivax* and P. *Falciparum*)) or those exhibiting an abnormally high burden (Enteric fever, and Pyrexia of Unknown Origin (PUO)) as per the state IDSP cell.
- 2. Population data: Punjab’s district-wise projected population data was used per the government’s estimates derived using the Component and Mathematical Method.[31] The data sources used for the projections included the 2011 Census and Sample Registration System (SRS). SRS provides time series data on fertility and mortality, which has been used for predicting their future levels.
- 3. GIS data: The digital version of the district maps of Punjab were retrieved from GitHub (https://github.com/datameet/maps/tree/master/Districts), which is shared under the Creative Commons Attribution 2.5 India license [32]. We projected the map in WGS 1984 UTM zone 43N for spatial analysis. qGIS software was used for mapping the FI hotspots in various districts of Punjab.

### Study variables

The number of FI cases due to Dengue, Chikungunya, Malaria (P.*Vivax*, P.*Falciparum*), Enteric fever, and PUO were the primary dependent variables. The week-wise FI diseases were retrieved for each district and were later compiled for the state for weeks and years, included in the study period. At the same time, factors that affect the interaction between human vectors like Total Population, Population Density, Place of residence (rural, urban), Gender (male, female), Occupation (Total workers, main workers, marginal workers, non-workers) were the critical, independent variables retrieved from the census [33].

### Statistical analysis

The annual trends and burden of FI were quantified and depicted as the number of cases and Incidence per 1,00,000 projected population. A comprehensive spatial autocorrelation and cluster analysis were conducted based on the FI disease burden incidence in different districts over the study period to discern the geographical clustering of FI between the districts. Given the extensive study periods and multiple data points, the anticipation of some missing data was addressed by planning to omit variables with substantial missing data from the dataset, while minor missed entries were imputed using their nearest neighbors. This approach aimed to avoid the introduction of errors in local spatial statistics that could arise from imputing global average, median, or mode values.

The identification of clusters or hot spots was initiated using the Getis-Ord Gi* statistic, or simply G*, based on incidence and average FI data values in a geographic area. This statistic contrasts the local mean of disease burden with the global mean, considering the spatial arrangement of the burden. It delineates hotspots where a specific location and its adjacent areas have values notably higher than the overall average value and cold spots where the local average is significantly lower than the global average [34, 35].

The formula for Getis-Ord G* is given as:

$${G}_{i}^{*}=\frac{\sum_{j=1}^{n}{w}_{i,j}{x}_{j }-\overline{X }\sum_{j=1}^{n}{w}_{i,j} }{S\sqrt{\frac{\sqrt{\left[n\sum_{j=1}^{n}{w}_{i,j}^{2}-{\left(\sum_{j=1}^{n}{w}_{i,j}\right)}^{2}\right]}}{n-1}}}$$

In the equation, X is the mean of all the values, and S is the standard deviation. The *x*_*j*_ is the value of the attribute at *j*^*th*^ location *w*_*ij*_ is the value of spatial weight attributed to the *i* and *j* location. The spatial weights matrix is kept same for all methods and is described subsequently. All these calculations were done using the Hotspot Analysis plugin of QGIS software. The identified clusters were further verified using the Spatial autocorrelation of the FI dataset, as it allows us to find patterns in complex data due to its multi-directional and multi-dimensional properties. The analysis was done using Moran’s I method, determining if the Incidence or crude disease burden in one district is influenced by nearby districts [36]. If this correlation exists, the data points aren't truly independent, violating basic statistical assumptions and potentially rendering many statistical tests unreliable. The values of the generated correlation coefficient can range between -1 to 1. However, while other coefficients measure perfect correlation to no correlation, Moran’s I differ (due to the more complex spatial calculations) from other correlation coefficients that estimate the correlation in binary form.

There are two types of Moran’s I statistics. We have first calculated Global Moran’s I to assess the overall dispersion of disease incidence in the state, which is denoted by the equation:

$$I=\frac{{\Sigma }_{i}{\Sigma }_{\dot{J}}{w}_{ij}{z}_{i} \frac{{z}_{j}}{{S}_{0}}}{\frac{{\Sigma }_{i}{z}_{\dot{i}}^{2}}{n}}$$

Assuming $\overline{\mathrm{X} }$ is the average incidence of cases of a particular disease in Punjab in a year and ${x}_{i}$ is the incidence in district *i*, and ${z}_{i}$ is the measure of the difference between the district’s incidence and the global average, i.e. $\left({x}_{i}-\overline{x }\right)$ is also called spatial lag, ${S}_{^\circ }$ while is the sum of all spatial weights which is elaborated as:

$${S}_{^\circ }= \sum i\sum j {w}_{ij}$$

Following this, we performed a more rigorous analysis in each district using Local Moran’s I to identify the spatial clustering of each disease, which is given by the equation.

$${I}_{i}=\frac{{\Sigma }_{j}{w}_{ij }{z}_{i }{z}_{i}}{{\Sigma }_{i}{z}_{i}^{2}}$$

Queen’s criterion was used to assign the same weight to the neighbors by counting the number of adjacent locations [37]. This matrix is row standardized, i.e., the sum of all weights given to neighbors of each district is 1. Moran’s I statistic tries to reject the null hypothesis of spatial randomness. Hence, there should be a significance factor (*p*-value) associated with each value, indicating the chances of observing it without having any spatial correlation. The p-values are calculated based on the methodology described by Anselin L [38]. The Local Moran’s I help classify areas based on local spatial patterns as:

- High-High (HH): An area with a high value is surrounded by areas with high values and indicates a hotspot
- Low-Low (LL): An area with a low value is surrounded by areas with low values and is labeled as a cold spot
- High-Low (HL): An area with a high value is surrounded by low-value areas. This indicates a spatial outlier.
- Low–High (LH): An area with a low value is surrounded by areas with high value, indicating a spatial outlier
- Not significant: The observed spatial pattern is not statistically significant, meaning that the observed pattern might be random and not coherent with the underlying spatial process.

Following this, we assessed the dispersion of FI**.** The primary goal of this analysis was to understand the central tendency of diseases throughout the state. This helped us understand how the number of cases varies in a district in these years and whether we can identify some patterns. Based on the results of the dispersion patterns, Negative Binomial Regression was applied to determine the association between spatiotemporal and population variables as per the census estimates using Python version 3.8.10. In negative binomial regression, *y* (average number of cases) is determined by a set of *k* regressor variables (the x’s). For *i*^th^ observation, the expression relating these quantities is

$$Total\;Cases\;of\;Dengue\;=\;Intercept\;+\;exp(\beta1\ast Pop.Density\;+\;\;\beta2\;\ast\;Total\;Female\;Pop....)$$

The regression coefficients *β1, β2, …, βk* are unknown parameters signifying the relative importance of a variable and are estimated from our disease data, *exp()* is an exponential function. We also excluded correlated variables (total population, male population, total worker population, main worker population, and non-worker population) as they were inflating/deflating the coefficients of other variables. During this analysis, we noticed some important details per the census data were missing, specifically for District Fazilka, as it was announced as the 21^st^ district of Punjab only after the last census in July 2011. We imputed these missing values as only a few were missing. For this, a weighted average of the available demographic information from those neighboring districts were calculated, which means that the proximity and population density of the neighboring districts would directly contribute to the estimates of Fazilka. This analysis was done using a special tool in Python called the '*geopandas*' library. We then used the population of each district to give more importance to districts with larger populations while calculating the average.

### Ethical clearance

Approved by IEC, AIIMS Bathinda, IEC/AIIMS/BTI/016, dated 29/01/2021 (IEC No: IEC-01/2020–012). Permissions were sought from DGHS Punjab regarding the access to data sets wide letter no. IDSP/ NHM/ Pb/19/4368, Dated: 23/12/2019.

## Results

### Disease burden and incidence

**Fig. 2:**
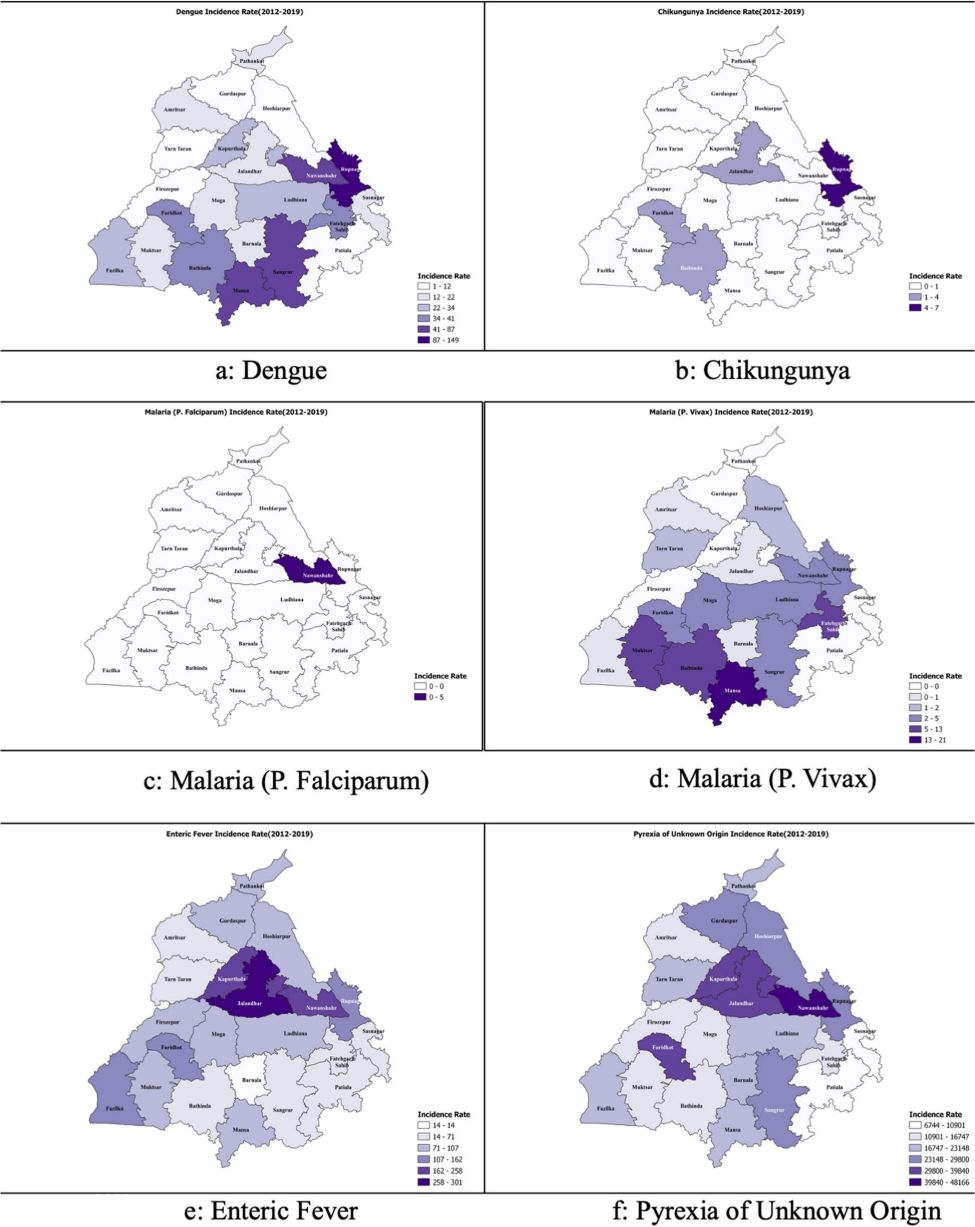
District-wise average Incidence rates of Febrile illnesses reported under IDSP Punjab between 2012–19

Tables 1, 2, and Fig. 2 summarised the disease burden of the FI diseases reported under IDSP and included in the study. Dengue varied, peaking in 2015 and 2017. Chikungunya remained sporadic, with a surge in the year 2016–17. Malaria patterns differed, as P. *falciparum* dipped till 2016, then depicted a resurgence, while P. *Vivax* was stable throughout. Enteric fever was consistent, highest in 2012, followed by a declining trend until 2019. PUO depicted the highest burden and was steady. Likewise, the incidence of reported FI varied, and PUO was most commonly reported, followed by Enteric fever, Dengue, and Malaria. The highest estimated incidence of confirmed Dengue, Chikungunya, and Enteric fever was 43.0, 5.3, and 116.0 cases per 100,000 population, while P. *Falciparum* and P. *Vivax* depicted the highest incidence of 0.5 and 6.2 during different years under study.

**Table 1:** Trends depicting the absolute numbers and incidence of different Febrile illnesses reported through IDSP platform in Punjab between 2012–19

Year	Population of Punjab^b^	Dengue		Chikungunya		Malaria (P. *Falciparum*)		Malaria (P. *Vivax*)		Enteric fever		Pyrexia of Unknown Origin	
		**Number of cases**	**Incidence**^**a**^	**Number of cases**	**Incidence**^**a**^	**Number of cases**	**Incidence**^**a**^	**Number of cases**	**Incidence**^**a**^	**Number of cases**	**Incidence**^**a**^	**Number of cases**	**Incidence**^**a**^
2012	28,023,000	883	3.2	0	0.0	76	0.3	1750	6.2	32,517	116.0	932,169	3326.4
2013	28,302,000	3692	13.0	0	0.0	147	0.5	1589	5.6	28,833	101.9	790,843	2794.3
2014	28,581,000	450	1.6	4	0.0	12	0.0	845	3.0	25,406	88.9	682,807	2389.0
2015	28,861,000	12,420	43.0	0	0.0	3	0.0	437	1.5	26,701	92.5	651,008	2255.7
2016	29,140,000	8179	28.1	1540	5.3	5	0.0	576	2.0	25,343	87.0	672,345	2307.3
2017	29,380,000	12,169	41.4	162	0.6	19	0.1	445	1.5	29,077	99.0	740,969	2522.0
2018	29,619,000	11,421	38.6	7	0.0	5	0.0	312	1.1	29,244	98.7	792,067	2674.2
2019	29,859,000	9889	33.1	8	0.0	22	0.1	632	2.1	32,999	110.5	781,212	2616.3

^a^Incidence/100,000 Population

^b^projected population of Punjab as per Census of India 2011-Population Projections For India And States 2011 – 2036

**Table 2:** Burden of major diseases contributing to the Febrile illnesses reported under IDSP in the state of Punjab between 2012–2019

**Year**	**Dengue**		**Chikungunya**		**Malaria (P. *Falciparum*)**		**Malaria (P. *Vivax*)**		**Enteric fever**		**Pyrexia of Unknown Origin**	
	**Mean ± SD**	**Min—Max**	**Mean ± SD**	**Min—Max**	**Mean ± SD**	**Min—Max**	**Mean ± SD**	**Min—Max**	**Mean ± SD**	**Min—Max**	**Mean ± SD**	**Min—Max**
**2012**	17 ± 36.4	0–136	0 ± 0	0–0	0.9 ± 1.9	0–9	33.7 ± 38.0	0–152	625.3 ± 226.8	234–1102	17,926.3 ± 2299.6	13,413–22,584
**2013**	71.2 ± 142	0–595	0 ± 0	0–0	2.8 ± 11.2	0–77	30.4 ± 38.2	0–160	554.5 ± 175.8	184–844	15,208.5 ± 1961.9	9539–19,239
**2014**	8.9 ± 19.4	0–107	0.1 ± 0.6	0–4	0.2 ± 1.1	0–8	16.3 ± 18.1	0–71	488.6 ± 163.9	157–799	13,130.9 ± 1634.5	10,050–16733
**2015**	239.7 ± 420.1	0–1393	0 ± 0	0–0	0.1 ± 0.2	0–1	8.4 ± 10.7	0–51	509.6 ± 179.9	182–821	12,323.9 ± 2193.4	6641–15,705
**2016**	157.2 ± 278.6	0–924	29.6 ± 58.9	0–235	0.1 ± 0.3	0–1	11.1 ± 12.8	0–51	489.2 ± 170.5	172–781	12,929.7 ± 1744	9111–16,666
**2017**	234 ± 503	0–1916	3.1 ± 6.7	0–34	0.4 ± 1.2	0–6	8.6 ± 10.1	0–43	558.8 ± 185.2	240–915	14,249.4 ± 1832.8	9832–18,107
**2018**	219.6 ± 402.4	0–1425	0.2 ± 0.4	0–2	0.1 ± 0.4	0–2	6.0 ± 8.0	0–31	562.4 ± 151.5	223–904	15,232.1 ± 1820.9	11,052–22805
**2019**	171.9 ± 374.3	0–1518	0.2 ± 0.5	0–2	0.4 ± 1.8	0–12	12.2 ± 18.8	0–81	634.6 ± 204	219–1017	15,023.3 ± 1484.9	11,091–18081
***p*-value**	< 0.001		< 0.001		< 0.010		< 0.001		< 0.001		< 0.001

### Spatial analysis and clustering

**Fig. 3:**
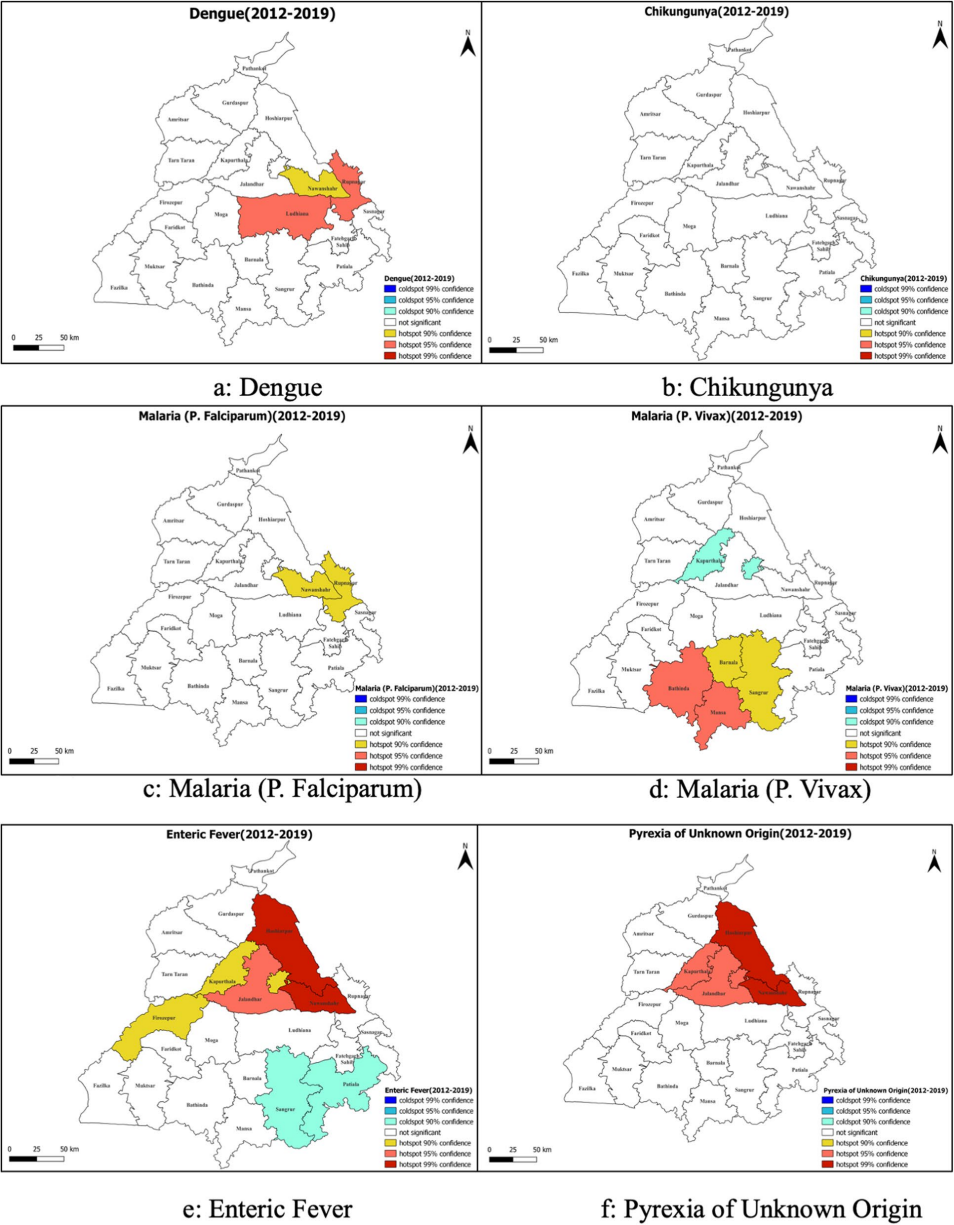
Getis-ord Gi* maps depicting the hotspots of incident febrile illnesses reported through IDSP in the state of Punjab (India) between 2012–19

Figure 3 and Supplementary Material 1 depict the clusters per the Getis-Ord Gi* method using the incidence rates and caseload from 2012–2019. Ludhiana and Rupnagar districts were strongly identified as significant hotspots for Dengue cases. Chikungunya cases depicted a uniform distribution, with no specific hotspots. For *P. Falciparum* malaria, Nawanshahr and Rupnagar emerged as less significant hotspots. Contrary to P. *Falciparum*, P. *Vivax* depicted hotspots in Bathinda, Mansa (95% Confidence). Jalandhar, Hoshiarpur, and Nawanshahr emerged as significant hot spots for enteric fever. Significant PUO hotspots were observed in Kapurthala, Jalandhar, Hoshiarpur, and Nawanshahr.

### Correlation and dispersion analysis

**Fig. 4:**
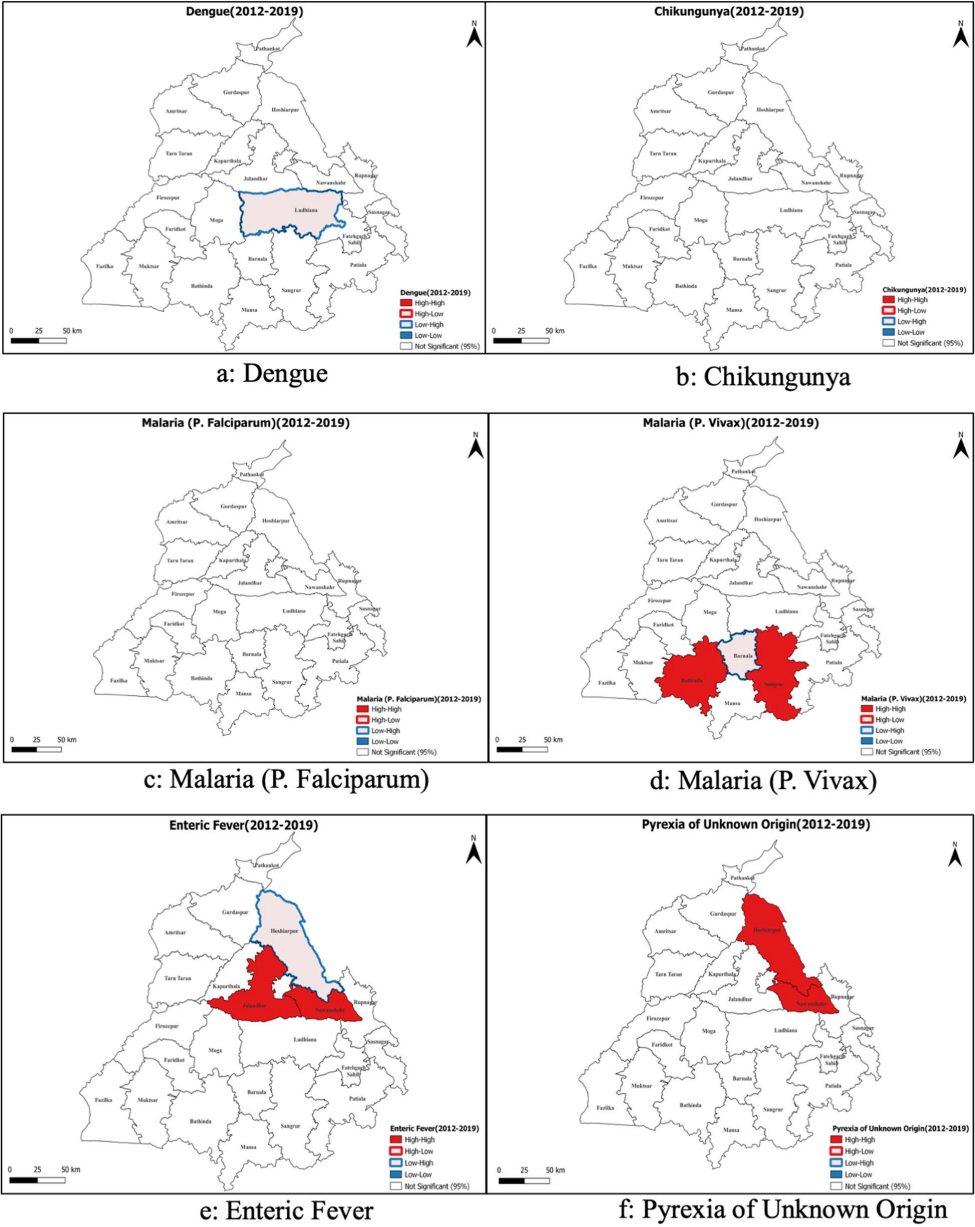
Moran’s I plot depicting the dispersion of incident febrile illnesses reported through IDSP in the state of Punjab (India) between 2012–19

**Fig. 5:**
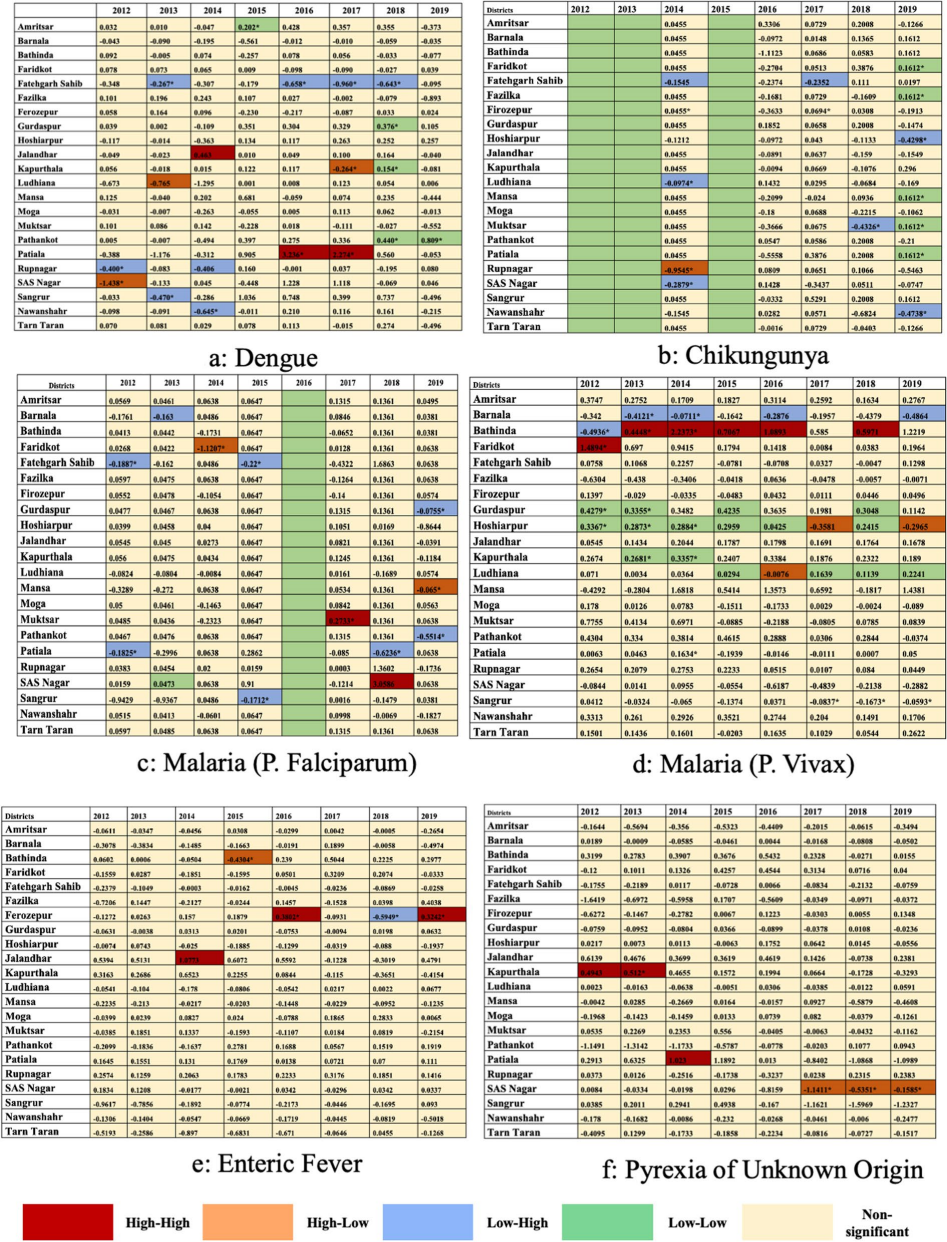
Heatmaps depicting the stable hotspots of febrile illnesses reported through IDSP in the state of Punjab (India) between 2012–19

**Fig. 6:**
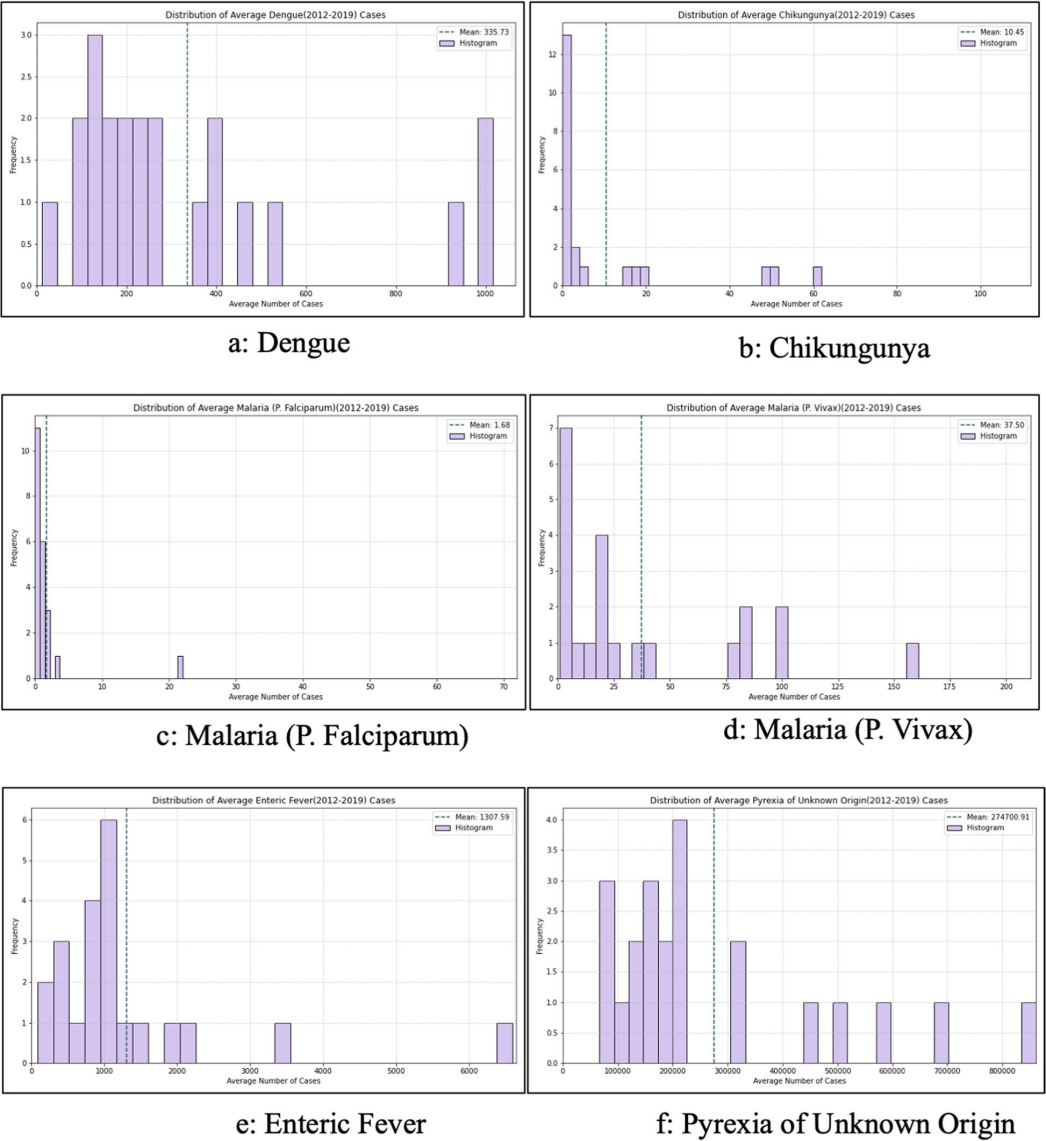
Bin Plots depicting the dispersion of various confirmed cases of febrile illnesses reported through IDSP in the state of Punjab (India) between 2012–19

Global Moran’s I analysis, verified using spatial autocorrelation and presented in Table 3, Supplementary Materials 2 and 3, revealed closer to 0 values for all diseases, indicating heterogeneous disease spread throughout the state. Dengue depicted negative Moran’s I values until 2014, indicating non-significant dispersion, contrary to the years 2016 and 2017. Likewise, Chikungunya depicted heterogeneity over the years, suggesting dispersion. However, P. *Falciparum* and *Vivax* depicted significant but infrequent clustering, but Enteric Fever and PUO’s Moran's I values were statistically non-significant. Visual maps rooted in Local Moran's I spatial autocorrelation further clarified the distribution patterns of diseases, identifying spatial outliers and regions with significant disease incidence (Fig. 4). Ludhiana was observed as a spatial outlier, and had a low incidence of Dengue but was surrounded by areas with higher incidence. However, no significant spatial autocorrelation patterns were observed for Chikungunya and P. *Falciparum* malaria. On the contrary, district Bathinda and Sangrur depicted a high incidence of P. *Vivax* malaria and was also surrounded by areas with high incidence, and district Barnala depicted a low value but was surrounded by areas with high values. Then, district Jalandhar and Nawanshahr depicted a high incidence of enteric fever and was surrounded by areas with high values, and district Hoshiarpur depicted low values but was surrounded by areas with high values. Lastly, Hoshiarpur and Nawanshahr depicted a high value and surrounding areas with a high incidence of PUO. Further, the district-wise Moran’s I values calculated individually for each FI between 2012–19 depict stable hotspots and Cold spots (Fig. 5, Supplementary material 4). Subsequently, the assessment of data dispersion through bin plots depicted a Poisson Distribution with indications of overdispersion for all diseases, as illustrated in Fig. 6.

**Table 3:** Spatial auto-correlation using Global Moran’s I of the febrile illness incidence reported under IDSP Punjab between 2012–2019

	**Dengue**		**Chikungunya**		**Malaria** **(P. *Falciparum*)**		**Malaria (P. *Vivax*)**		**Enteric Fever**		**Pyrexia of Unknown Origin**	
Year	**Moran's I**	***p*-value**	**Moran's I**	***p*-value**	**Moran's I**	***p*-value**	**Moran's I**	***p*-value**	**Moran's I**	***p*-value**	**Moran's I**	***p*-value**
2012	-0.136	0.06	-	0.**001**	-0.055	0.334	0.164	0.064	-0.111	0.3	-0.135	0.244
2013	-0.123	0.155	-	**0.001**	-0.056	0.319	0.133	0.066	-0.026	0.393	-0.038	0.467
2014	-0.159	0.098	-0.050	0.486	-0.050	0.478	0.386	**0.005**	0.013	0.29	-0.026	0.378
2015	0.106	0.092	-	**0.001**	0.091	**0.017**	0.135	**0.049**	-0.016	0.389	0.095	0.135
2016	0.282	**0.013**	-0.135	0.255	-	**0.001**	0.160	0.061	0.009	0.308	-0.033	0.433
2017	0.198	**0.033**	0.063	**0.038**	0.024	0.166	0.065	0.139	0.045	0.211	-0.130	0.248
2018	0.130	0.053	0.000	0.302	0.337	**0.006**	0.074	0.1	-0.021	0.391	-0.203	0.064
2019	-0.119	0.29	-0.055	0.442	-0.061	0.329	0.160	0.053	-0.009	0.355	-0.176	0.157

### Regression analysis and impact assessment

Based on dispersion, the negative binomial regression was applied to elucidate the influence of demographic variables on the occurrence of FI (Table 4). All FI except Chikungunya and Malaria due to P. Falciparum depicted a significant occurrence at baseline as seen in intercept values (*p*-value < 0.05). However, none of the studied independent variables depicted any effect on the occurrence of any disease, except the impact of the marginal working population on Chikungunya and P. Falciparum, but with a negligible coefficient value.

**Table 4:** Population variables predicting stable hotspots in Punjab using a Negative Binomial regression equation

	**Dengue**		**Chikungunya**		**Malaria (P. *Falciparum*)**		**Malaria (P. *Vivax*)**		**Enteric fever**		**Pyrexia of Unknown Origin**	
	**Coefficients**	***p*-value**	**Coefficients**	***p*-value**	**Coefficients**	***p*-value**	**Coefficients**	***p*-value**	**Coefficients**	***p*-value**	**Coefficients**	***p*-value**
Intercept	4.9996	** < 0.001**	-1.863	0.153	-0.2146	0.907	3.6063	**0.002**	6.009	** < 0.001**	8.4895	** < 0.001**
Population Density	0.0005	0.808	0.003	0.273	-0.0022	0.551	-0.0002	0.923	0.0005	0.811	0.0022	0.306
**Gender**												
Total Female Population	0.0000	0.676	0.0000	0.954	0.0000	0.553	0.0000	0.641	0.000	0.302	0.0000	0.544
**Residence**												
Rural Population	0.0000	0.706	0.0000	0.988	0.0000	0.381	0.0000	0.737	0.000	0.320	0.0000	0.582
Urban Population	0.0000	0.619	0.0000	0.946	0.0000	0.470	0.0000	0.613	0.000	0.319	0.0000	0.527
**Occupation**												
Marginal working population	0.0000	0.701	0.0000	**0.012**	-0.0001	**0.038**	0.0000	0.106	0.000	0.819	0.0000	0.796

## Discussion

This is among the few papers from India that have presented such an analysis using the routinely collected IDSP data. Our study yielded the following key findings. *First,* PUO depicted the highest incidence among all the FI, while Malaria had the lowest annual incidence. We observed a rising incidence of Dengue and Enteric fever, while Chikungunya depicted an occasional spike during the study period. *Second,* there were significant inter-district variations in the burden of all FI. The FI expressed clustering at the start of the study period, with more dispersion in the latter part. P. Vivax was seen with high incidence in southern districts of Punjab, especially Bathinda. Enteric Fever incidence is high in central and north-eastern districts, with Nawanshahr and Jalandhar being significant hotspots of the disease. Hoshiarpur and Nawanshahr are also hotspots of PUO during the duration of the study. *Lastly,* the number of cases in each district has shown over-dispersion for each disease with little dependence on population characteristics.

The rising trends of FI observed in our study are coherent with the reports from Low-middle-income and high-income countries [39]. Two distinct trends can explain these escalated estimates: increasing vector-borne disease incidence and interspersed, infrequent outbreaks attributed to a widening spectrum of domestic and imported VBD. This rising trend can be attributed to changes in human activities like trade and travel, rising urbanization, a rise in population, and increasing encroachment of wildlands. These activities have also increased anthropogenic greenhouse gases, leading to abrupt climatic alterations that can affect the mechanics of disease transmission, geographic spread, and re-emergence of VBD through multiple pathways [40, 41]. Also, climatic change directly affects vector species and their ecosystem (including urban habitats), in which vectors may or may not survive. Because the vectors are poikilothermic, global warming will further increase the vector's abundance, survival, and feeding activity, and in similar proportions to the pathogen's development rate [42].

The state is amongst the top five states of India in terms of Dengue burden and also suffered a Chikungunya outbreak around 2016 [12]. For Dengue, Fatehgarh Sahib was identified as a consistent "Low–High" outlier over the years, indicating that this district, with a low value, is surrounded by districts with higher values. There were no stable hotspots, coldspots, or "High-Low" outliers for Dengue. Chikungunya did not exhibit any stable spatial patterns, and no districts were identified as consistent hotspots, cold spots, or outliers, similar to an analysis from Barbados [11]. The two are arboviral diseases, and two principal vector species, Aedes *aegypti,* and Aedes *albopictus*, are known for transmission, and outbreaks can be mitigated commonly [43]. Dengue and Chikungunya control also calls for advocacy and health awareness campaigns as the vector. While Dengue is largely endemic to the region, the Chikungunya outbreak was uncommon until the last decade. The first outbreak was recorded in 1963 in Calcutta, but the disease re-emerged in India in 2005 [44]. There have been Chikungunya outbreaks in other parts of Northern India between 2013–17, similar to what was observed in Punjab [13, 45]. Previous studies demonstrated that age-specific seroprevalence has been uniform across age groups in this region, suggesting an epidemic transmission pattern, susceptibility of the population to the virus, and the absence of herd immunity [46]. In the absence of established human-to-human transmission, the infrequent spikes can be attributed to the introduction of the mutated strains of the virus through extensive human movements, as seen in other countries [47]. The Chikungunya outbreaks in North India during the study period were mainly attributed to the Indian Ocean Lineage of the East-Central South African CHIKV genotype, which increased the adaptability of Chikungunya virus to Aedes *albopictus* mosquitoes [48]. Such mutations increase the ability of the Chikungunya virus to adapt to a new vector and expand its geographical distribution, making it a dynamic pathogen of global public health concern [49]. With a common vector, improved Dengue and Chikungunya control can be achieved through reliable epidemic forecasting systems that detect temporal anomalies in disease incidence [50]. The mosquito mates, feeds, rests, and lays eggs in and around urban human habitation. So, getting infected with Dengue and Chikungunya is due to inappropriate public health measures around the patient only. Control of vectors is warranted by source reduction elimination of container habitats that are favorable for vector breeding [51].

P. *Falciparum* malaria depicted no stable spatial patterns, with no districts emerging as hotspots, cold spots, or outliers. But for P. *Vivax*, Bathinda stood out as a stable hot spot, suggesting a high disease incidence in this district and its neighboring areas. On the other hand, Hoshiarpur was identified as a stable cold spot, indicating a consistently low disease incidence compared to its neighboring districts. No outliers were observed for P. *Vivax*. The incidence of Malaria due to P. *Falciparum* was observed to be < 1 case/1000 population at risk, while it was a little higher for P. *Vivax* and is therefore categorized in category one, where the states/UTs have the Annual Parasite Incidence of less than one and all the districts in the state with API less than one., and thus it can be considered that the state is already on the lines to eliminate Malaria within the timelines stipulated by National Framework for Malaria Elimination in India (2016–2030) [30]. More effective control strategies that affect entomological and epidemiological endpoints are required. Google trends have been effective in identifying outbreaks early and helping control them effectively [13].

The estimates of Enteric fever were as high as 116 cases/lakh population and are slightly lower than recent modeled estimates from India [18, 52]. It was also the most widely spread disease, with about 5 districts depicting hotspots, but exhibited no stable spatial patterns, with no districts consistently identified as hotspots, coldspots, or outliers. Enteric fever is majorly influenced by the Water and Sanitation Hygiene (WASH) indicators that depend on the social determinants of health [53]. Such a high burden also necessitates the introduction of typhoid vaccine for children under five to prevent unwarranted morbidity and mortality [17]. Similarly, the PUO was the most common type of FI reported in Punjab, but no districts consistently showed any form of spatial autocorrelation, whether it be hotspots, coldspots, or outliers. The absence of distinct hot or cold regions suggests that interventions may need a broader, more generalized approach for PUO. The high burden of PUO also calls for health strengthening, as the most commonly cited factors associated with PUO diagnosis include the year of evaluation, physician experience, quality of referral center, and the clinical characteristics of the fever itself [20, 54]. Apart from the regional socioeconomic factors, such as healthcare access and variations in practice patterns, geographical factors may also play a significant role [55]. The diagnosis of PUO is complex. It is elaborated as a persistent fever above 38.3 °C (100°F) that remains undiagnosed for at least three weeks, of which one week should be investigated following hospitalization. Due to inadequate diagnostic facilities, primary care physicians tend to label patients with PUO if they are negative for available laboratory tests. However, previous studies have reported that various infections, including Tuberculosis, are responsible for only 43%-53% of PUO cases, and that too in the hospital setting [56, 57]. High prevalence of PUO points toward other infectious diseases prevalent in the area, like bacteremia, scrub typhus, leptospirosis, or miss-diagnosis of the known causes. This calls for a review of the management practices of health professionals regarding communicable diseases.

We observed that Nawanshahr was a common incidence hotspot for Dengue, Malaria (P. Falciparum), Enteric fever, and PUO, followed by Rupnagar, which was a common hotspot for Dengue, Malaria (P. Falciparum), and Enteric Fever, and pose a risk of outbreaks in neighboring districts as well. Spatial mapping of the districts and identifying hot spots could help policymakers and program officers identify districts that require more focus. A rapid review of literature in the Indian health context revealed that the GIS tool had been extensively used to control vector-borne diseases, outbreaks, and disease surveillance [28, 58–60]. These studies have identified hot spots for prioritizing public health interventions. The significant inter-district variations can be attributed to vector abundance, geographical determinants like transitional swamps and unmanaged pasture proportions, and population demographic composition [26]. The availability of water bodies (natural in rural and artificial in urban areas) significantly affected the clustering of FI. Our study reinforces the hypothesis developed by previous studies that Malaria and Dengue tend to cluster with specific geographic units significantly [61]. Our study calls for augmented actions in such districts, and stricter implementation of public health interventions can impact the overall indicators of the state. Thus, spatial heterogeneity is common in several health issues, including FI, vector-borne diseases, malnutrition, etc. Against the background of these spatial mapping exercises, we recommend a GIS visualization platform with regular real-time data updation under the IDSP to monitor various illnesses so that it is highlighted if a localized spurt of disease cases occurs.

The study's specific strengths and limitations need to be acknowledged. District-wise data collected for eight years using standard case definitions helped us build robust estimates about the disease trends and hotspots. The results emanating from the study will be crucial in designing future disease containment measures. A rigorous statistical analysis makes the results reliable and reasonably valid. Some important caveats to the data used are essential to note during interpreting results. It was an ecological study, and we did not have patient-level data. In addition, the absence of access to environmental variables such as climate change, population growth, vector density, and patient-level data prevented us from making a more robust model supporting our hypothesis related to hot spots. ﻿We also cannot comment upon the accuracy of the disease reporting system as some cases might be due to the inaccessibility of health systems. We could not include the patients diagnosed and treated in the private sector.

To conclude, the present study demonstrated the stable hot spots for certain FI reported under IDSP and their relation with the demographic composition. Specific policy implications are emerging from the study. We have observed hot spots for most of the studies FI. This can significantly push the VBD control program already in place. Identifying the spatial clusters of infection is crucial for health planning and re-distributing the available resources as and when required. The present study demonstrates that information obtained through IDSP can describe the spatial epidemiology of FI at crude spatial scales. Still, our only challenge for the next few years is to develop effective interventions that allow real-time identification of the local spatial heterogeneity using existing surveillance data and are affordable simultaneously.

### Supplementary Information

**Additional file 1.**

**Additional file 2.**

**Additional file 3.**

**Additional file 4.**

## Data Availability

The IDSP state-level datasets analyzed during the current study are not publicly available and were retrieved from the state government upon special request, but are available from the corresponding author on reasonable request.
