# Supplementary material for: Mapping the stability of febrile illness hotspots in Punjab from 2012 to 2019- a spatial clustering and regression analysis
Source: BMC Public Health. 2023 Oct 16;23:2014. doi: 10.1186/s12889-023-16930-y (PMC10580620; doi:10.1186/s12889-023-16930-y)
Supplement: Supplementary file 1 — Additional file 1. [file 12889_2023_16930_MOESM1_ESM.pdf]

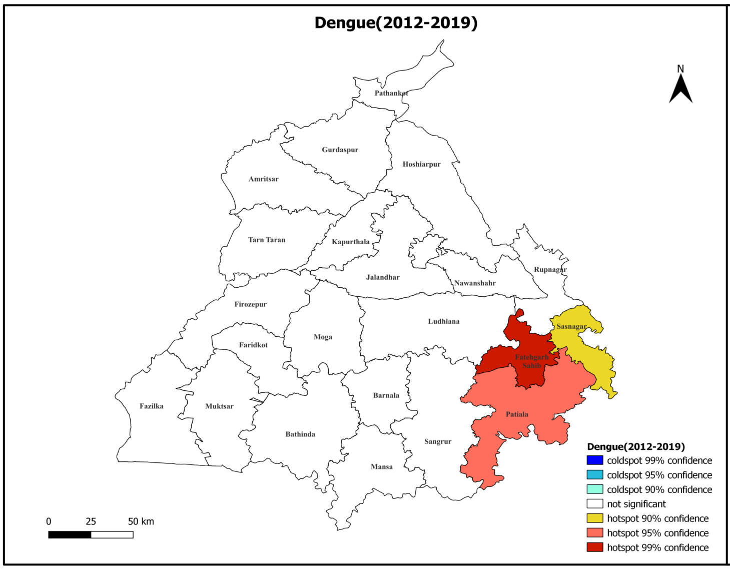

S1a: Dengue

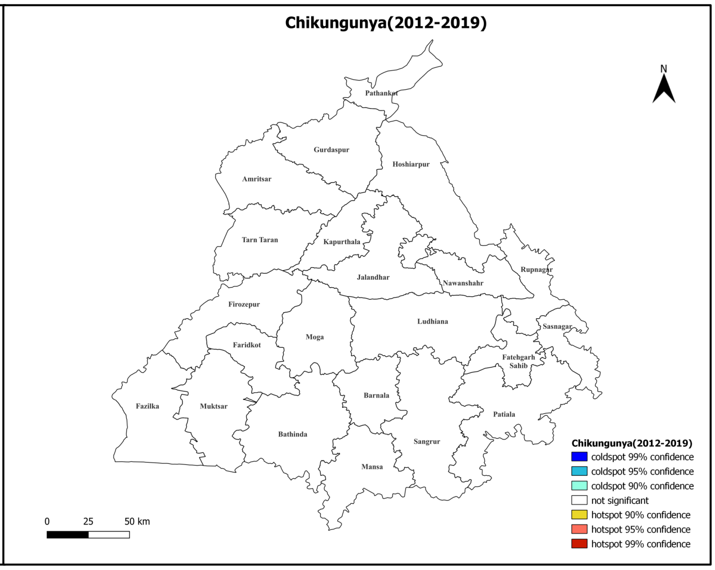

S1b: Chikungunya

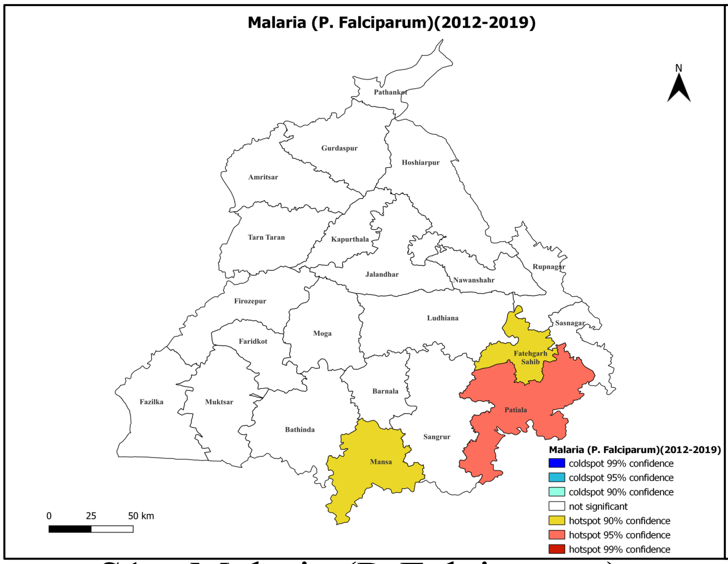

S1c: Malaria (P. Falciparum)

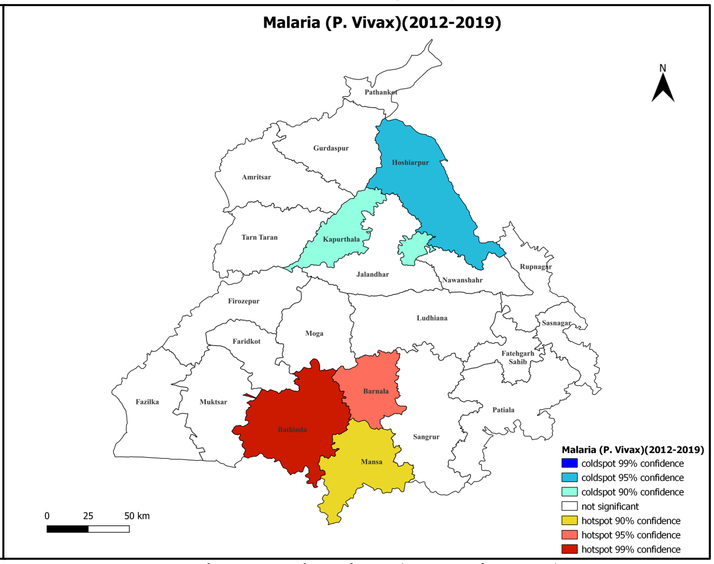

S1d: Malaria (P. Vivax)

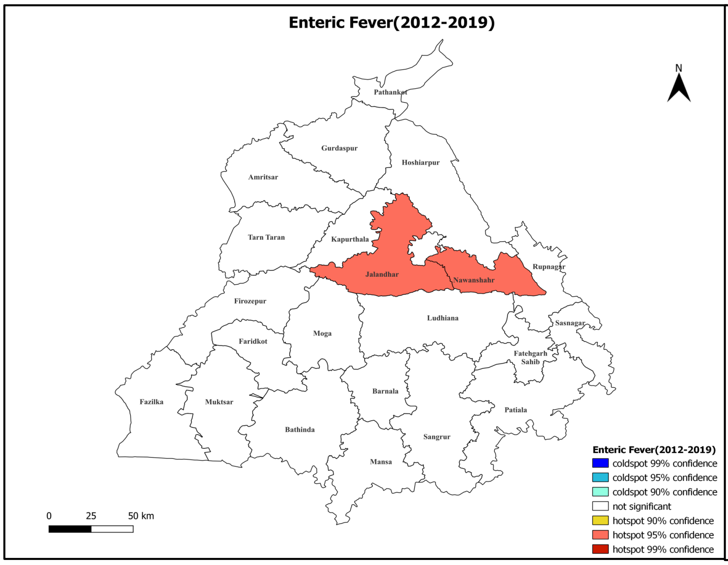

S1 e: Enteric Fever

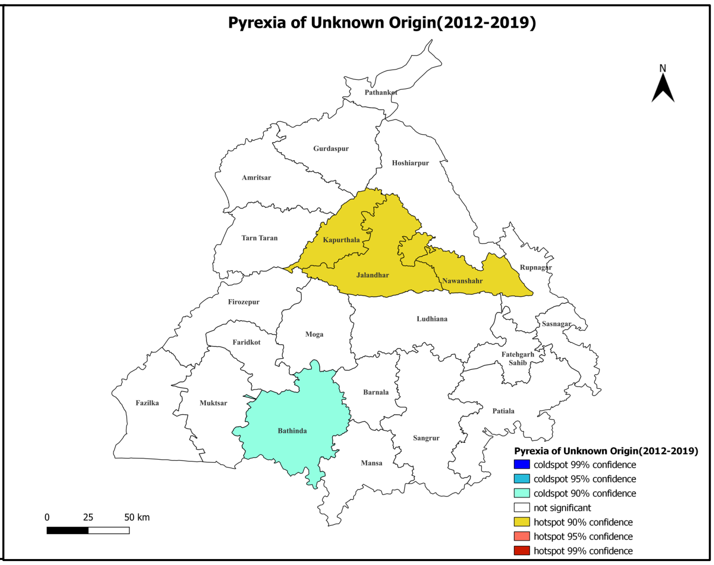

S1 f: Pyrexia of Unknown Origin

Supplementary Material S1 (a-f): Getis-ord Gi* maps depicting the caseload hotspots of different febrile illnesses reported through IDSP in the state of Punjab (India) between 2012-19
