# Supplementary material for: Mapping the stability of febrile illness hotspots in Punjab from 2012 to 2019- a spatial clustering and regression analysis
Source: BMC Public Health. 2023 Oct 16;23:2014. doi: 10.1186/s12889-023-16930-y (PMC10580620; doi:10.1186/s12889-023-16930-y)
Supplement: Supplementary file 2 — Additional file 2. [file 12889_2023_16930_MOESM2_ESM.pdf]

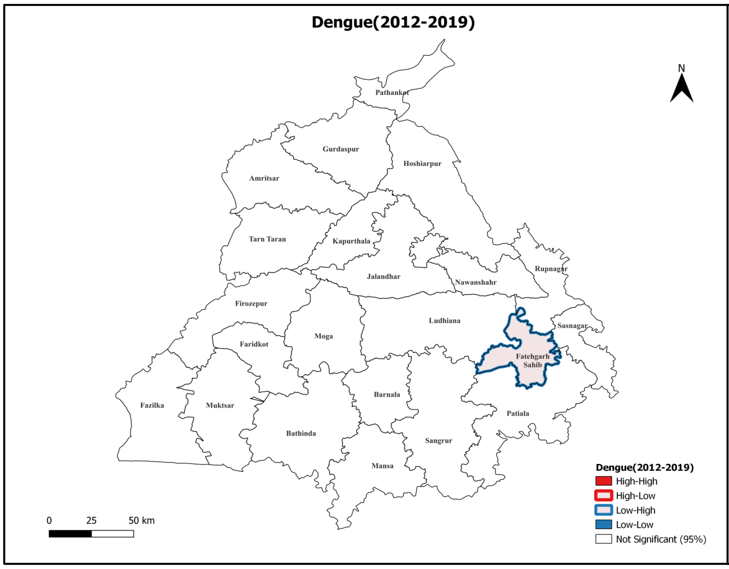

S2 a: Dengue

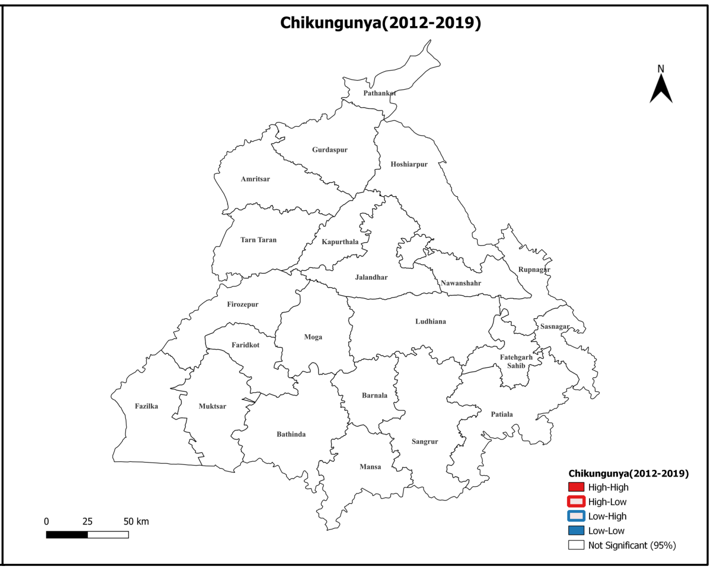

S2 b: Chikungunya

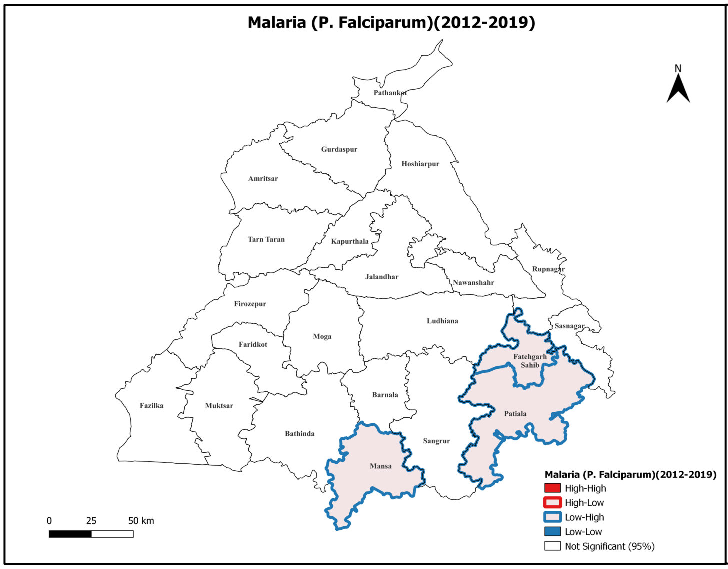

S2 c: Malaria (P. Falciparum)

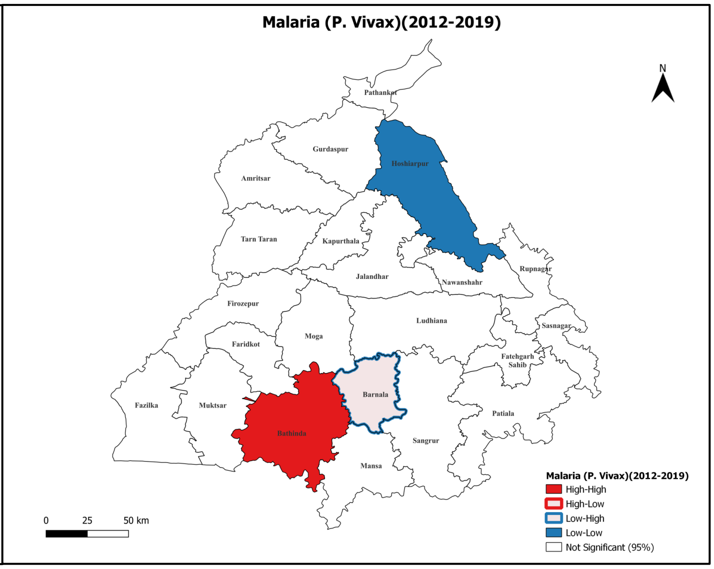

S2 d: Malaria (P. Vivax)

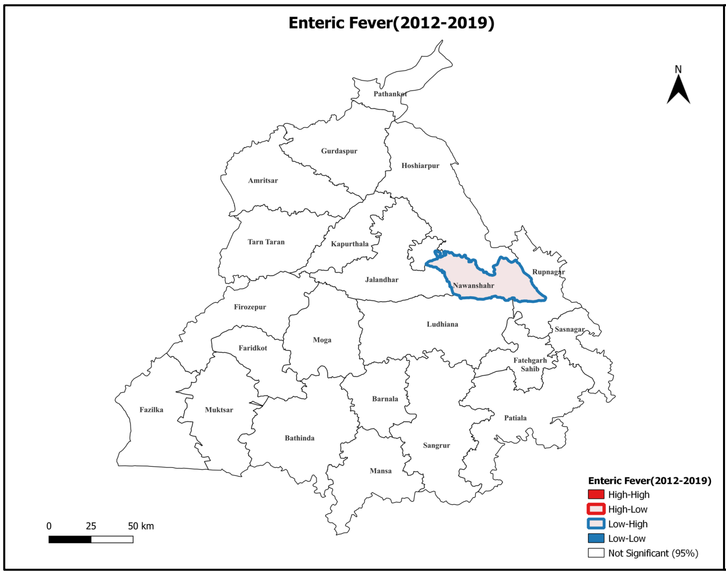

S2 e: Enteric Fever

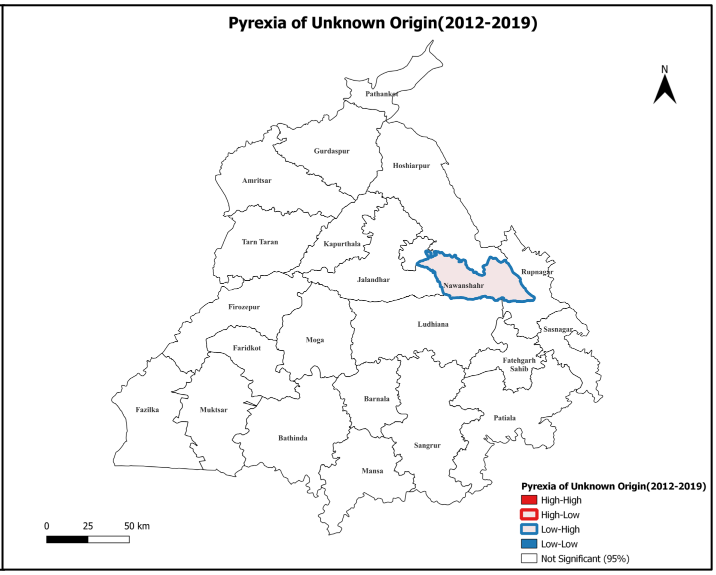

S2 f: Pyrexia of Unknown Origin

Supplementary Material S2(a-f): Moran's I plot depicting the dispersion of average confirmed cases of febrile illnesses reported through IDSP in the state of Punjab (India) between 2012-19
