# Supplementary material for: Mapping the stability of febrile illness hotspots in Punjab from 2012 to 2019- a spatial clustering and regression analysis
Source: BMC Public Health. 2023 Oct 16;23:2014. doi: 10.1186/s12889-023-16930-y (PMC10580620; doi:10.1186/s12889-023-16930-y)
Supplement: Supplementary file 3 — Additional file 3. [file 12889_2023_16930_MOESM3_ESM.pdf]

Supplementary material 3: Spatial auto-correlation of the febrile illnesses reported under IDSP Punjab between 2012-2019 using Global Moran's I.

Year	Dengue		Chikungunya		Malaria (P. *Falciparum*)		Malaria (P. *Vivax*)		Enteric fever		Pyrexia of Unknown Origin	
	Moran's I	p-value	Moran's I	p-value	Moran's I	p-value	Moran's I	p-value	Moran's I	p-value	Moran's I	p-value
**2012**	-0.139	0.069	-	-	-0.054	0.448	0.037	0.25	-0.004	0.346	-0.031	0.417
**2013**	-0.161	0.019	-	-	-0.044	0.46	0.037	0.231	0.032	0.161	-0.043	0.449
**2014**	-0.095	0.255	-0.050	0.495	-0.073	0.441	0.210	0.031	0.033	0.191	-0.021	0.366
**2015**	-0.125	0.277	-	-	0.225	0.016	-0.059	0.487	-0.011	0.318	0.054	0.213
**2016**	0.169	0.04	-0.136	0.265	-	-	-0.095	0.395	0.018	0.19	0.094	0.136
**2017**	0.055	0.18	0.022	0.051	-0.013	0.323	-0.099	0.361	0.045	0.19	0.025	0.291
**2018**	0.093	0.099	0.022	0.258	0.366	0.006	-0.006	0.325	-0.037	0.414	-0.095	0.408
**2019**	-0.130	0.252	-0.052	0.456	-0.047	0.474	0.043	0.198	-0.014	0.29	-0.045	0.422
