# Supplementary material for: Mapping the stability of febrile illness hotspots in Punjab from 2012 to 2019- a spatial clustering and regression analysis
Source: BMC Public Health. 2023 Oct 16;23:2014. doi: 10.1186/s12889-023-16930-y (PMC10580620; doi:10.1186/s12889-023-16930-y)
Supplement: Supplementary file 4 — Additional file 4. [file 12889_2023_16930_MOESM4_ESM.pdf]

Supplementary material 4

Supplementary material 4a: District-wise trends of Local Moran's I value to depict the stability of the hotspots for the Dengue cases reported under IDSP in Punjab between 2012-19

	2012			2013			2014			2015			2016			2017			2018			2019		
District	Moran's I	p-value	Classification	Moran's I	p-value	Classification	Moran's I	p-value	Classification	Moran's I	p-value	Classification	Moran's I	p-value	Classification	Moran's I	p-value	Classification	Moran's I	p-value	Classification	Moran's I	p-value	Classification
**Amritsar**	0.0316	0.271	NS	0.0098	0.357	NS	-0.05	0.48	NS	0.2022	0.047	L-L	0.43	0.056	NS	0.3572	0.227	NS	0.3548	0.066	NS	-0.3727	0.281	NS
**Barnala**	-0.043	0.344	NS	-0.0903	0.258	NS	-0.2	0.238	NS	-0.5613	0.06	NS	-0.01	0.364	NS	-0.0097	0.484	NS	-0.0591	0.305	NS	-0.0354	0.253	NS
**Bathinda**	0.0924	0.166	NS	-0.0052	0.381	NS	0.074	0.129	NS	-0.2572	0.457	NS	0.08	0.368	NS	0.0555	0.329	NS	-0.0327	0.279	NS	-0.0771	0.153	NS
**Faridkot**	0.0777	0.434	NS	0.0731	0.307	NS	0.065	0.213	NS	0.0094	0.3	NS	-0.1	0.399	NS	-0.0901	0.317	NS	-0.0268	0.414	NS	0.0385	0.499	NS
**Fatehgarh Sahib**	-0.3475	0.052	NS	-0.2668	0.033	L-H	-0.31	0.124	NS	-0.1786	0.051	NS	-0.66	0.002	L-H	-0.9603	0.01	L-H	-0.6431	0.016	L-H	-0.0954	0.288	NS
**Fazilka**	0.1013	0.405	NS	0.1962	0.227	NS	0.243	0.303	NS	0.1068	0.478	NS	0.03	0.392	NS	-0.0018	0.421	NS	-0.0787	0.299	NS	-0.8933	0.472	NS
**Ferozepur**	0.0577	0.182	NS	0.1635	0.092	NS	0.096	0.31	NS	-0.2304	0.071	NS	-0.22	0.197	NS	-0.0872	0.304	NS	0.0328	0.352	NS	0.0241	0.334	NS
**Gurdaspur**	0.0385	0.355	NS	0.0021	0.354	NS	-0.11	0.217	NS	0.3506	0.128	NS	0.3	0.127	NS	0.3293	0.164	NS	0.3756	0.015	L-L	0.1053	0.416	NS
**Hoshiarpur**	-0.117	0.146	NS	-0.0144	0.255	NS	-0.36	0.45	NS	0.134	0.066	NS	0.12	0.075	NS	0.2632	0.166	NS	0.2521	0.109	NS	0.257	0.218	NS
**Jalandhar**	-0.0491	0.287	NS	-0.0226	0.348	NS	0.463	0.042	H-H	0.0096	0.438	NS	0.05	0.299	NS	0.0999	0.228	NS	0.1637	0.054	NS	-0.0396	0.433	NS
**Kapurthala**	0.0555	0.457	NS	-0.018	0.313	NS	0.015	0.303	NS	0.1223	0.198	NS	0.12	0.103	NS	-0.2638	0.035	H-L	0.1544	0.001	L-L	-0.0808	0.329	NS
**Ludhiana**	-0.6726	0.063	NS	-0.7647	0.005	H-L	-1.3	0.173	NS	0.0007	0.452	NS	0.01	0.459	NS	0.1226	0.147	NS	0.0538	0.415	NS	0.0061	0.349	NS
**Mansa**	0.1246	0.294	NS	-0.0396	0.341	NS	0.202	0.214	NS	0.6806	0.096	NS	-0.06	0.245	NS	0.074	0.327	NS	0.2345	0.214	NS	-0.4442	0.17	NS
**Moga**	-0.0309	0.348	NS	-0.0066	0.42	NS	-0.26	0.119	NS	-0.0547	0.308	NS	0.01	0.471	NS	0.1127	0.335	NS	0.0616	0.34	NS	-0.0132	0.394	NS
**Muktsar**	0.1007	0.488	NS	0.0858	0.307	NS	0.142	0.28	NS	-0.2276	0.281	NS	0.02	0.471	NS	-0.1106	0.318	NS	-0.0274	0.407	NS	-0.5524	0.087	NS
**Pathankot**	0.0051	0.191	NS	-0.007	0.234	NS	-0.49	0.125	NS	0.3974	0.208	NS	0.27	0.311	NS	0.3357	0.062	NS	0.44	0.014	L-L	0.8088	0.035	L-L
**Patiala**	-0.3879	0.099	NS	-1.1762	0.422	NS	-0.31	0.182	NS	0.9048	0.142	NS	3.24	0.009	H-H	2.2743	0.014	H-H	0.5595	0.139	NS	-0.0527	0.235	NS
**Rupnagar**	-0.4003	0.009	L-H	-0.0833	0.305	NS	-0.41	0.038	L-H	0.1603	0.293	NS	-0	0.497	NS	0.037	0.439	NS	-0.1947	0.057	NS	0.08	0.378	NS
**SAS Nagar**	-1.4378	0.001	H-L	-0.133	0.096	NS	0.045	0.456	NS	-0.4476	0.115	NS	1.23	0.103	NS	1.1177	0.129	NS	-0.0688	0.055	NS	0.0461	0.272	NS
**Sangrur**	-0.0326	0.406	NS	-0.47	0.017	L-H	-0.29	0.135	NS	1.0364	0.051	NS	0.75	0.104	NS	0.3985	0.178	NS	0.7373	0.071	NS	-0.4962	0.336	NS
**Nawanshahr**	-0.0981	0.252	NS	-0.0906	0.285	NS	-0.64	0.005	L-H	-0.0112	0.219	NS	0.21	0.246	NS	0.1159	0.245	NS	0.1606	0.201	NS	-0.2154	0.28	NS
**Tarn Taran**	0.0698	0.373	NS	0.0812	0.431	NS	0.029	0.393	NS	0.0779	0.463	NS	0.11	0.483	NS	-0.0154	0.445	NS	0.2736	0.242	NS	-0.4955	0.198	NS

**NS: Non-significant; L-L: Low-Low; L-H: low-High; H-L: High Low; H-H: High-High*

Supplementary material 4b: District wise trends of Local Moran’s I value to depict the stability of the hotspots for the Chikungunya cases reported under IDSP in Punjab between 2012-19

	2012			2013			2014			2015			2016			2017			2018			2019		
	Moran's I	p-value	Classification	Moran's I	p-value	Classification	Moran's I	p-value	Classification	Moran's I	p-value	Classification	Moran's I	p-value	Classification	Moran's I	p-value	Classification	Moran's I	p-value	Classification	Moran's I	p-value	Classification
**Amritsar**		0.001	L-L		0.001	L-L	0.0455	0.14	NS		0.001	L-L	0.3306	0.071	NS	0.0729	0.324	NS	0.2008	0.401	NS	-0.1266	0.158	NS
**Barnala**		0.001	L-L		0.001	L-L	0.0455	0.234	NS		0.001	L-L	-0.0972	0.37	NS	0.0148	0.369	NS	0.1365	0.291	NS	0.1612	0.298	NS
**Bathinda**		0.001	L-L		0.001	L-L	0.0455	0.234	NS		0.001	L-L	-1.1123	0.161	NS	0.0686	0.36	NS	0.0583	0.311	NS	0.1612	0.298	NS
**Faridkot**		0.001	L-L		0.001	L-L	0.0455	0.195	NS		0.001	L-L	-0.2704	0.097	NS	0.0513	0.283	NS	0.3876	0.261	NS	0.1612	0.001	L-L
**Fatehgarh Sahib**		0.001	L-L		0.001	L-L	-0.1545	0.001	L-H		0.001	L-L	-0.2374	0.18	NS	-0.2352	0.047	L-H	0.111	0.375	NS	0.0197	0.5	NS
**Fazilka**		0.001	L-L		0.001	L-L	0.0455	0.095	NS		0.001	L-L	-0.1681	0.246	NS	0.0729	0.48	NS	-0.1609	0.211	NS	0.1612	0.001	L-L
**Ferozepur**		0.001	L-L		0.001	L-L	0.0455	0.337	NS		0.001	L-L	-0.3633	0.272	NS	0.0694	0.226	NS	0.0308	0.491	NS	-0.1913	0.111	NS
**Gurdaspur**		0.001	L-L		0.001	L-L	0.0455	0.195	NS		0.001	L-L	0.1852	0.291	NS	0.0658	0.442	NS	0.2008	0.295	NS	-0.1474	0.19	NS
**Hoshiarpur**		0.001	L-L		0.001	L-L	-0.1212	0.054	NS		0.001	L-L	-0.0972	0.28	NS	0.043	0.336	NS	-0.1133	0.281	NS	-0.4298	0.001	L-H
**Jalandhar**		0.001	L-L		0.001	L-L	0.0455	0.287	NS		0.001	L-L	-0.0891	0.473	NS	0.0637	0.432	NS	-0.159	0.19	NS	-0.1549	0.27	NS
**Kapurthala**		0.001	L-L		0.001	L-L	0.0455	0.337	NS		0.001	L-L	-0.0094	0.482	NS	0.0669	0.377	NS	-0.1076	0.248	NS	0.296	0.335	NS
**Ludhiana**		0.001	L-L		0.001	L-L	-0.0974	0.04	L-H		0.001	L-L	0.1432	0.245	NS	0.0295	0.47	NS	-0.0684	0.336	NS	-0.169	0.108	NS
**Mansa**		0.001	L-L		0.001	L-L	0.0455	0.14	NS		0.001	L-L	-0.2099	0.208	NS	-0.024	0.251	NS	0.0936	0.486	NS	0.1612	0.001	L-L
**Moga**		0.001	L-L		0.001	L-L	0.0455	0.287	NS		0.001	L-L	-0.18	0.117	NS	0.0688	0.274	NS	-0.2215	0.105	NS	-0.1062	0.258	NS
**Muktsar**		0.001	L-L		0.001	L-L	0.0455	0.195	NS		0.001	L-L	-0.3666	0.125	NS	0.0675	0.457	NS	-0.4326	0.038	L-H	0.1612	0.001	L-L
**Pathankot**		0.001	L-L		0.001	L-L	0.0455	0.095	NS		0.001	L-L	0.0547	0.414	NS	0.0586	0.212	NS	0.2008	0.438	NS	-0.21	0.29	NS
**Patiala**		0.001	L-L		0.001	L-L	0.0455	0.14	NS		0.001	L-L	-0.5558	0.422	NS	0.3876	0.15	NS	0.2008	0.402	NS	0.1612	0.001	L-L
**Rupnagar**		0.001	L-L		0.001	L-L	-0.9545	0.001	H-L		0.001	L-L	0.0809	0.345	NS	0.0651	0.474	NS	0.1066	0.396	NS	-0.5463	0.421	NS
**SAS Nagar**		0.001	L-L		0.001	L-L	-0.2879	0.001	L-H		0.001	L-L	0.1428	0.109	NS	-0.3437	0.138	NS	0.0511	0.427	NS	-0.0747	0.283	NS
**Sangrur**		0.001	L-L		0.001	L-L	0.0455	0.222	NS		0.001	L-L	-0.0332	0.345	NS	0.5291	0.231	NS	0.2008	0.221	NS	0.1612	0.314	NS
**Nawanshahr**		0.001	L-L		0.001	L-L	-0.1545	0.222	NS		0.001	L-L	0.0282	0.492	NS	0.0571	0.488	NS	-0.6824	0.474	NS	-0.4738	0.001	L-H
**Tarn Taran**		0.001	L-L		0.001	L-L	0.0455	0.138	NS		0.001	L-L	-0.0016	0.399	NS	0.0729	0.327	NS	-0.0403	0.315	NS	-0.1266	0.173	NS

**NS: Non-significant; L-L: Low-Low; L-H: low-High; H-L: High Low; H-H: High-High*

Supplementary material 4c: District-wise trends of Local Moran's I value to depict the stability of the hotspots for the Malaria (P. *Falciparum*)cases reported under IDSP in Punjab between 2012-19

	**2012**			**2013**			**2014**			**2015**			**2016**			**2017**			**2018**			**2019**		
District	Moran's I	p_value	Classification	Moran's I	p_value	Classification	Moran's I	p_value	Classification	Moran's I	p_value	Classification	Moran's I	p_value	Classification	Moran's I	p_value	Classification	Moran's I	p_value	Classification	Moran's I	p_value	Classification
**Amritsar**	0.0569	0.201	NS	0.0461	0.134	NS	0.0638	0.419	NS	0.0647	0.25	NS		0.001	L-L	0.1315	0.358	NS	0.1361	0.389	NS	0.0495	0.39	NS
**Barnala**	-0.1761	0.137	NS	-0.163	0.024	L-H	0.0486	0.464	NS	0.0647	0.406	NS		0.001	L-L	0.0846	0.413	NS	0.1361	0.408	NS	0.0381	0.317	NS
**Bathinda**	0.0413	0.367	NS	0.0442	0.404	NS	-0.1731	0.099	NS	0.0647	0.406	NS		0.001	L-L	-0.0652	0.451	NS	0.1361	0.408	NS	0.0381	0.317	NS
**Faridkot**	0.0268	0.331	NS	0.0422	0.422	NS	-1.1207	0.001	H-L	0.0647	0.326	NS		0.001	L-L	0.0128	0.347	NS	0.1361	0.493	NS	0.0638	0.407	NS
**Fatehgarh Sahib**	-0.1887	0.002	L-H	-0.162	0.061	NS	0.0486	0.448	NS	-0.22	0.001	L-H		0.001	L-L	-0.4322	0.417	NS	1.6863	0.054	NS	0.0638	0.299	NS
**Fazilka**	0.0597	0.324	NS	0.0475	0.499	NS	0.0638	0.284	NS	0.0647	0.175	NS		0.001	L-L	-0.1264	0.107	NS	0.1361	0.292	NS	0.0638	0.343	NS
**Ferozepur**	0.0552	0.129	NS	0.0478	0.22	NS	-0.1054	0.317	NS	0.0647	0.445	NS		0.001	L-L	-0.14	0.141	NS	0.1361	0.257	NS	0.0574	0.284	NS
**Gurdaspur**	0.0477	0.325	NS	0.0467	0.249	NS	0.0638	0.485	NS	0.0647	0.326	NS		0.001	L-L	0.1315	0.259	NS	0.1361	0.498	NS	-0.0755	0.047	L-H
**Hoshiarpur**	0.0399	0.144	NS	0.0458	0.225	NS	0.04	0.338	NS	0.0647	0.477	NS		0.001	L-L	0.1051	0.241	NS	0.0169	0.487	NS	-0.8644	0.286	NS
**Jalandhar**	0.0545	0.196	NS	0.045	0.405	NS	0.0273	0.274	NS	0.0647	0.477	NS		0.001	L-L	0.0821	0.158	NS	0.1361	0.333	NS	-0.0391	0.148	NS
**Kapurthala**	0.056	0.088	NS	0.0475	0.305	NS	0.0434	0.424	NS	0.0647	0.445	NS		0.001	L-L	0.1245	0.107	NS	0.1361	0.257	NS	-0.1184	0.115	NS
**Ludhiana**	-0.0824	0.282	NS	-0.0804	0.243	NS	-0.0084	0.428	NS	0.0647	0.445	NS		0.001	L-L	0.0161	0.453	NS	-0.1689	0.101	NS	0.0574	0.266	NS
**Mansa**	-0.3289	0.062	NS	-0.272	0.069	NS	0.0638	0.38	NS	0.0647	0.25	NS		0.001	L-L	0.0534	0.39	NS	0.1361	0.399	NS	-0.065	0.001	H-L
**Moga**	0.05	0.484	NS	0.0461	0.351	NS	-0.1463	0.079	NS	0.0647	0.477	NS		0.001	L-L	0.0842	0.394	NS	0.1361	0.333	NS	0.0563	0.449	NS
**Muktsar**	0.0485	0.398	NS	0.0436	0.487	NS	-0.2323	0.067	NS	0.0647	0.326	NS		0.001	L-L	0.2733	0.035	H-H	0.1361	0.498	NS	0.0638	0.405	NS
**Pathankot**	0.0467	0.375	NS	0.0476	0.442	NS	0.0638	0.271	NS	0.0647	0.175	NS		0.001	L-L	0.1315	0.491	NS	0.1361	0.292	NS	-0.5514	0.009	L-H
**Patiala**	-0.1825	0.02	L-H	-0.2996	0.093	NS	0.0638	0.38	NS	0.2862	0.052	NS		0.001	L-L	-0.085	0.19	NS	-0.6236	0.001	L-H	0.0638	0.486	NS
**Rupnagar**	0.0383	0.311	NS	0.0454	0.274	NS	0.02	0.229	NS	0.0159	0.224	NS		0.001	L-L	0.0003	0.38	NS	1.3602	0.051	NS	-0.1736	0.132	NS
**SAS Nagar**	0.0159	0.256	NS	0.0473	0.001	L-L	0.0638	0.38	NS	0.91	0.129	NS		0.001	L-L	-0.1214	0.162	NS	3.0586	0.001	H-H	0.0638	0.486	NS
**Sangrur**	-0.9429	0.396	NS	-0.9367	0.196	NS	0.0486	0.448	NS	-0.1712	0.037	L-H		0.001	L-L	0.0016	0.388	NS	-0.1479	0.291	NS	0.0381	0.322	NS
**Nawanshahr**	0.0515	0.407	NS	0.0413	0.387	NS	-0.0601	0.401	NS	0.0647	0.421	NS		0.001	L-L	0.0998	0.311	NS	-0.0069	0.491	NS	-0.1827	0.069	NS
**Tarn Taran**	0.0597	0.163	NS	0.0485	0.233	NS	0.0638	0.368	NS	0.0647	0.255	NS		0.001	L-L	0.1315	0.334	NS	0.1361	0.401	NS	0.0638	0.486	NS

**NS: Non-significant; L-L: Low-Low; L-H: low-High; H-L: High Low; H-H: High-High*

Supplementary material 4d: District-wise trends of Local Moran's I value to depict the stability of the hotspots for the Malaria *(P. Vivax)*cases reported under IDSP in Punjab between 2012-19

	2012			2013			2014			2015			2016			2017			2018			2019		
District	Moran's I	p_value	Classification	Moran's I	p_value	Classification	Moran's I	p_value	Classification	Moran's I	p_value	Classification	Moran's I	p_value	Classification	Moran's I	p_value	Classification	Moran's I	p_value	Classification	Moran's I	p_value	2019 Classification
**Amritsar**	0.3747	0.085	NS	0.2752	0.111	NS	0.1709	0.111	NS	0.1827	0.204	NS	0.3114	0.076	NS	0.2592	0.087	NS	0.1634	0.173	NS	0.2767	0.087	NS
**Barnala**	-0.342	0.126	NS	-0.4121	0.033	L-H	-0.0711	0.023	L-H	-0.1642	0.068	NS	-0.2876	0.023	L-H	-0.1957	0.057	NS	-0.4379	0.071	NS	-0.4864	0.037	L-H
**Bathinda**	-0.4936	0.002	L-H	0.4448	0.001	H-H	2.2373	0.001	H-H	0.7067	0.024	H-H	1.0893	0.029	H-H	0.585	0.093	NS	0.5971	0.028	H-H	1.2219	0.062	NS
**Faridkot**	1.4894	0.039	H-H	0.697	0.171	NS	0.9415	0.068	NS	0.1794	0.24	NS	0.1418	0.301	NS	0.0084	0.307	NS	0.0383	0.252	NS	0.1964	0.245	NS
**Fatehgarh Sahib**	0.0758	0.413	NS	0.1068	0.36	NS	0.2257	0.263	NS	-0.0781	0.387	NS	-0.0708	0.368	NS	0.0327	0.451	NS	-0.0047	0.381	NS	0.1298	0.346	NS
**Fazilka**	-0.6304	0.144	NS	-0.438	0.138	NS	-0.3406	0.266	NS	-0.0418	0.391	NS	0.0636	0.464	NS	-0.0478	0.399	NS	-0.0057	0.357	NS	-0.0071	0.444	NS
**Ferozepur**	0.1397	0.081	NS	-0.029	0.319	NS	-0.0335	0.41	NS	-0.0483	0.359	NS	0.0432	0.37	NS	0.0111	0.278	NS	0.0446	0.428	NS	0.0496	0.302	NS
**Gurdaspur**	0.4279	0.029	L-L	0.3355	0.019	L-L	0.3482	0.075	NS	0.4235	0.032	L-L	0.3635	0.058	NS	0.1981	0.25	NS	0.3048	0.036	L-L	0.1142	0.337	NS
**Hoshiarpur**	0.3367	0.004	L-L	0.2873	0.004	L-L	0.2884	0.002	L-L	0.2959	0.001	L-L	0.0425	0.001	L-L	-0.3581	0.001	H-L	0.2415	0.01	L-L	-0.2965	0.023	H-L
**Jalandhar**	0.0545	0.33	NS	0.1434	0.231	NS	0.2044	0.197	NS	0.1787	0.133	NS	0.1798	0.124	NS	0.1691	0.196	NS	0.1764	0.055	NS	0.1678	0.187	NS
**Kapurthala**	0.2674	0.072	NS	0.2681	0.02	L-L	0.3357	0.038	L-L	0.2407	0.101	NS	0.3384	0.058	NS	0.1876	0.11	NS	0.2322	0.065	NS	0.189	0.116	NS
**Ludhiana**	0.071	0.232	NS	0.0034	0.115	NS	0.0364	0.159	NS	0.0294	0.022	L-L	-0.0076	0.037	H-L	0.1639	0.008	L-L	0.1139	0.018	L-L	0.2241	0.007	L-L
**Mansa**	-0.4292	0.438	NS	-0.2804	0.332	NS	1.6818	0.052	NS	0.5414	0.182	NS	1.3573	0.055	NS	0.6592	0.117	NS	-0.1817	0.273	NS	1.4381	0.098	NS
**Moga**	0.178	0.183	NS	0.0126	0.372	NS	0.0783	0.172	NS	-0.1511	0.223	NS	-0.1733	0.19	NS	0.0029	0.443	NS	-0.0024	0.412	NS	-0.089	0.363	NS
**Muktsar**	0.7755	0.09	NS	0.4134	0.228	NS	0.6971	0.114	NS	-0.0885	0.156	NS	-0.2188	0.093	NS	-0.0805	0.216	NS	0.0785	0.224	NS	0.0839	0.108	NS
**Pathankot**	0.4304	0.136	NS	0.334	0.143	NS	0.3814	0.185	NS	0.4615	0.126	NS	0.2888	0.367	NS	0.0306	0.413	NS	0.2844	0.09	NS	-0.0374	0.288	NS
**Patiala**	0.0063	0.446	NS	0.0463	0.462	NS	0.1634	0.355	NS	-0.1939	0.469	NS	-0.0146	0.337	NS	-0.0111	0.338	NS	0.0007	0.336	NS	0.05	0.436	NS
**Rupnagar**	0.2654	0.125	NS	0.2079	0.199	NS	0.2753	0.173	NS	0.2233	0.216	NS	0.0515	0.449	NS	0.0107	0.415	NS	0.084	0.433	NS	0.0449	0.467	NS
**SAS Nagar**	-0.0844	0.205	NS	0.0141	0.156	NS	0.0955	0.058	NS	-0.0554	0.47	NS	-0.6187	0.121	NS	-0.4839	0.167	NS	-0.2138	0.418	NS	-0.2882	0.137	NS
**Sangrur**	0.0412	0.362	NS	-0.0324	0.164	NS	-0.065	0.207	NS	-0.1374	0.068	NS	0.0371	0.139	NS	-0.0837	0.2	NS	-0.1673	0.166	NS	-0.0593	0.271	NS
**Nawanshahr**	0.3313	0.075	NS	0.261	0.081	NS	0.2926	0.08	NS	0.3521	0.058	NS	0.2744	0.145	NS	0.204	0.206	NS	0.1491	0.053	NS	0.1706	0.252	NS
**Tarn Taran**	0.1501	0.337	NS	0.1436	0.168	NS	0.1601	0.217	NS	-0.0203	0.411	NS	0.1635	0.12	NS	0.1029	0.217	NS	0.0544	0.202	NS	0.2622	0.206	NS

**NS: Non-significant; L-L: Low-Low; L-H: low-High; H-L: High Low; H-H: High-High*

Supplementary material 4e: District-wise trends of Local Moran's I value to depict the stability of the hotspots for the Enteric Fever cases reported under IDSP in Punjab between 2012-19

	2012			2013			2014			2015			2016			2017			2018			2019		
District	Moran's I	p_value	Classification	Moran's I	p_value	Classification	Moran's I	p_value	Classification	Moran's I	p_value	Classification	Moran's I	p_value	Classification	Moran's I	p_value	Classification	Moran's I	p_value	Classification	Moran's I	p_value	Classification
**Amritsar**	-0.0611	0.349	NS	-0.0347	0.338	NS	-0.0456	0.256	NS	0.0308	0.496	NS	-0.0299	0.471	NS	0.0042	0.466	NS	-0.0005	0.477	NS	-0.2654	0.328	NS
**Barnala**	-0.3078	0.183	NS	-0.3834	0.145	NS	-0.1485	0.381	NS	-0.1663	0.381	NS	-0.0191	0.467	NS	0.1899	0.313	NS	-0.0058	0.482	NS	-0.4974	0.241	NS
**Bathinda**	0.0602	0.118	NS	0.0006	0.12	NS	-0.0504	0.132	NS	-0.4304	0.048	H-L	0.239	0.152	NS	0.5044	0.108	NS	0.2225	0.269	NS	0.2977	0.225	NS
**Faridkot**	-0.1559	0.304	NS	0.0287	0.463	NS	-0.1851	0.353	NS	-0.1595	0.363	NS	0.0501	0.348	NS	0.3209	0.153	NS	0.2074	0.186	NS	-0.0333	0.495	NS
**Fatehgarh Sahib**	-0.2379	0.23	NS	-0.1049	0.231	NS	-0.0003	0.471	NS	-0.0162	0.491	NS	-0.0045	0.421	NS	-0.0236	0.398	NS	-0.0869	0.298	NS	-0.0258	0.19	NS
**Fazilka**	-0.7206	0.197	NS	0.1447	0.47	NS	-0.2127	0.364	NS	-0.0244	0.455	NS	0.1457	0.307	NS	-0.1528	0.349	NS	0.0398	0.404	NS	0.4038	0.122	NS
**Ferozepur**	-0.1272	0.438	NS	0.0263	0.374	NS	0.157	0.246	NS	0.1879	0.178	NS	0.3802	0.049	H-H	-0.0931	0.07	NS	-0.5949	0.036	L-H	0.3242	0.036	H-H
**Gurdaspur**	-0.0631	0.437	NS	-0.0038	0.437	NS	0.0313	0.388	NS	0.0201	0.462	NS	-0.0753	0.417	NS	-0.0094	0.46	NS	0.0198	0.494	NS	0.0632	0.417	NS
**Hoshiarpur**	-0.0074	0.36	NS	0.0743	0.166	NS	-0.025	0.089	NS	-0.1885	0.185	NS	-0.1299	0.139	NS	-0.0319	0.389	NS	-0.088	0.346	NS	-0.1937	0.31	NS
**Jalandhar**	0.5394	0.131	NS	0.5131	0.183	NS	1.0773	0.03	H-H	0.6072	0.099	NS	0.5592	0.12	NS	-0.1228	0.476	NS	-0.3019	0.381	NS	0.4791	0.147	NS
**Kapurthala**	0.3163	0.097	NS	0.2686	0.146	NS	0.6523	0.062	NS	0.2255	0.247	NS	0.0844	0.313	NS	-0.115	0.455	NS	-0.3651	0.243	NS	-0.4154	0.316	NS
**Ludhiana**	-0.0541	0.222	NS	-0.104	0.067	NS	-0.178	0.173	NS	-0.0806	0.246	NS	-0.0542	0.34	NS	0.0217	0.42	NS	0.0022	0.442	NS	0.0677	0.361	NS
**Mansa**	-0.2235	0.15	NS	-0.213	0.17	NS	-0.0217	0.406	NS	-0.0203	0.414	NS	-0.1448	0.381	NS	-0.0229	0.319	NS	-0.0952	0.357	NS	-0.1235	0.305	NS
**Moga**	-0.0399	0.2	NS	0.0239	0.278	NS	0.0827	0.259	NS	0.024	0.194	NS	-0.0788	0.318	NS	0.1865	0.174	NS	0.2833	0.119	NS	0.0065	0.455	NS
**Muktsar**	-0.0385	0.453	NS	0.1851	0.22	NS	0.1337	0.308	NS	-0.1593	0.31	NS	-0.1107	0.288	NS	0.0184	0.292	NS	0.0819	0.371	NS	-0.2154	0.355	NS
**Pathankot**	-0.2099	0.324	NS	-0.1836	0.259	NS	-0.1637	0.406	NS	0.2781	0.289	NS	0.1688	0.253	NS	0.0567	0.408	NS	0.1519	0.217	NS	0.1919	0.099	NS
**Patiala**	0.1645	0.189	NS	0.1551	0.157	NS	0.131	0.325	NS	0.1769	0.271	NS	0.0138	0.261	NS	0.0721	0.351	NS	0.07	0.361	NS	0.111	0.292	NS
**Rupnagar**	0.2574	0.157	NS	0.1259	0.327	NS	0.2063	0.233	NS	0.1783	0.317	NS	0.2233	0.29	NS	0.3176	0.195	NS	0.1851	0.145	NS	0.1416	0.215	NS
**SAS Nagar**	0.1834	0.277	NS	0.1208	0.411	NS	-0.0177	0.466	NS	-0.0021	0.441	NS	0.0342	0.317	NS	-0.0296	0.426	NS	0.0342	0.447	NS	0.0337	0.461	NS
**Sangrur**	-0.9617	0.275	NS	-0.7856	0.36	NS	-0.1892	0.42	NS	-0.0774	0.446	NS	-0.2173	0.414	NS	-0.0446	0.464	NS	-0.1695	0.479	NS	0.093	0.342	NS
**Nawanshahr**	-0.1306	0.238	NS	-0.1404	0.064	NS	-0.0547	0.076	NS	-0.0669	0.127	NS	-0.1719	0.069	NS	-0.0445	0.318	NS	-0.0819	0.25	NS	-0.5018	0.061	NS
**Tarn Taran**	-0.5193	0.07	NS	-0.2586	0.269	NS	-0.897	0.1	NS	-0.6831	0.189	NS	-0.671	0.153	NS	-0.0646	0.415	NS	0.0455	0.444	NS	-0.1268	0.186	NS

Supplementary material 4f: District-wise trends of Local Moran's I value to depict the stability of the hotspots for the Malaria *(P. Vivax)*cases reported under IDSP in Punjab between 2012-19

	2012			2013			2014			2015			2016			2017			2018			2019		
District	Moran's I	p_value	Classification	Moran's I	p_value	Classification	Moran's I	p_value	Classification	Moran's I	p_value	Classification	Moran's I	p_value	Classification	Moran's I	p_value	Classification	Moran's I	p_value	Classification	Moran's I	p_value	Classification
**Amritsar**	-0.1644	0.247	NS	-0.5694	0.16	NS	-0.356	0.179	NS	-0.5323	0.143	NS	-0.4409	0.302	NS	-0.2015	0.353	NS	-0.0615	0.384	NS	-0.3494	0.283	NS
**Barnala**	0.0189	0.367	NS	-0.0009	0.464	NS	-0.0585	0.39	NS	-0.0461	0.461	NS	0.0044	0.269	NS	-0.0168	0.353	NS	-0.0808	0.15	NS	-0.0502	0.171	NS
**Bathinda**	0.3199	0.138	NS	0.2783	0.156	NS	0.3907	0.143	NS	0.3676	0.199	NS	0.5432	0.076	NS	0.2328	0.214	NS	-0.0271	0.184	NS	0.0155	0.206	NS
**Faridkot**	-0.12	0.355	NS	0.1011	0.323	NS	0.1326	0.287	NS	0.4257	0.108	NS	0.4544	0.058	NS	0.3134	0.165	NS	0.0716	0.389	NS	0.04	0.43	NS
**Fatehgarh Sahib**	-0.1755	0.239	NS	-0.2189	0.21	NS	0.0117	0.159	NS	-0.0728	0.154	NS	0.0066	0.455	NS	-0.0834	0.453	NS	-0.2132	0.223	NS	-0.0759	0.351	NS
**Fazilka**	-1.6419	0.073	NS	-0.6972	0.318	NS	-0.5958	0.306	NS	0.1707	0.335	NS	-0.5609	0.332	NS	-0.0349	0.43	NS	-0.0971	0.385	NS	-0.0372	0.322	NS
**Ferozepur**	-0.6272	0.33	NS	-0.1467	0.426	NS	-0.2782	0.315	NS	0.0067	0.448	NS	0.1223	0.21	NS	-0.0303	0.182	NS	0.0055	0.285	NS	0.1348	0.094	NS
**Gurdaspur**	-0.0759	0.485	NS	-0.0952	0.395	NS	-0.0804	0.461	NS	0.0366	0.338	NS	-0.0899	0.233	NS	-0.0378	0.292	NS	0.0108	0.44	NS	-0.0236	0.446	NS
**Hoshiarpur**	0.0217	0.414	NS	0.0073	0.461	NS	0.0113	0.452	NS	-0.0063	0.454	NS	0.1752	0.273	NS	0.0642	0.335	NS	0.0145	0.493	NS	-0.0556	0.35	NS
**Jalandhar**	0.6139	0.063	NS	0.4676	0.122	NS	0.3699	0.141	NS	0.3619	0.137	NS	0.4619	0.128	NS	0.1426	0.316	NS	-0.0738	0.479	NS	0.2381	0.24	NS
**Kapurthala**	0.4943	0.024	H-H	0.512	0.035	H-H	0.4655	0.069	NS	0.1572	0.286	NS	0.1994	0.272	NS	0.0664	0.36	NS	-0.1728	0.382	NS	-0.3293	0.389	NS
**Ludhiana**	0.0023	0.496	NS	-0.0163	0.493	NS	-0.0638	0.301	NS	-0.0051	0.469	NS	0.0306	0.38	NS	-0.0385	0.421	NS	-0.0122	0.273	NS	0.0591	0.268	NS
**Mansa**	-0.0042	0.429	NS	0.0285	0.339	NS	-0.2669	0.205	NS	0.0164	0.358	NS	-0.0157	0.472	NS	0.0927	0.183	NS	-0.5879	0.067	NS	-0.4608	0.077	NS
**Moga**	-0.1968	0.179	NS	-0.1423	0.352	NS	-0.1459	0.354	NS	0.0133	0.458	NS	0.0739	0.343	NS	0.082	0.334	NS	-0.0379	0.428	NS	-0.1261	0.259	NS
**Muktsar**	0.0535	0.435	NS	0.2269	0.173	NS	0.2353	0.16	NS	0.556	0.112	NS	-0.0405	0.475	NS	-0.0063	0.367	NS	-0.0432	0.295	NS	-0.1162	0.269	NS
**Pathankot**	-1.1491	0.156	NS	-1.3142	0.17	NS	-1.1733	0.176	NS	-0.5787	0.236	NS	-0.0778	0.347	NS	-0.0203	0.392	NS	0.1077	0.416	NS	0.0943	0.295	NS
**Patiala**	0.2913	0.238	NS	0.6325	0.177	NS	1.023	0.044	H-H	1.1892	0.078	NS	0.013	0.154	NS	-0.8402	0.08	NS	-1.0868	0.064	NS	-1.0989	0.092	NS
**Rupnagar**	0.0373	0.453	NS	0.0126	0.425	NS	-0.2516	0.353	NS	-0.1738	0.455	NS	-0.3237	0.362	NS	0.0238	0.432	NS	0.2315	0.302	NS	0.2383	0.282	NS
**SAS Nagar**	0.0084	0.394	NS	-0.0334	0.44	NS	-0.0198	0.486	NS	0.0296	0.432	NS	-0.8159	0.121	NS	-1.1411	0.001	H-L	-0.5351	0.004	H-L	-0.1585	0.006	H-L
**Sangrur**	0.0385	0.377	NS	0.2011	0.313	NS	0.2941	0.253	NS	0.4938	0.125	NS	-0.167	0.403	NS	-1.1621	0.155	NS	-1.5969	0.117	NS	-1.2327	0.16	NS
**Nawanshahr**	-0.178	0.175	NS	-0.1682	0.161	NS	-0.0086	0.258	NS	-0.232	0.185	NS	-0.0268	0.164	NS	-0.0461	0.267	NS	-0.006	0.439	NS	-0.2477	0.189	NS
**Tarn Taran**	-0.4095	0.053	NS	0.1299	0.174	NS	-0.1733	0.143	NS	-0.1858	0.209	NS	-0.2234	0.238	NS	-0.0816	0.362	NS	-0.0727	0.352	NS	-0.1517	0.25	NS

**NS: Non-significant; L-L: Low-Low; L-H: low-High; H-L: High Low; H-H: High-High*
